# Research on Shaft-Rate Magnetic Field Detection Method for Underwater Targets Based on Differential High-Order Dual-Coupled Duffing Oscillators

**DOI:** 10.3390/s26175545

**Published:** 2026-08-31

**Authors:** Yong Yang, Litian Zhang, Chao Zuo, Jihui He, Dezhi Cao, Chengran Liu, Hailin Qiu, Xiaobing Zhang, Xiaofei Yang, Jun Ouyang

**Affiliations:** 1 Naval University of Engineering, Wuhan 430033, China; 2 School of Integrated Circuits, Huazhong University of Science and Technology, Wuhan 430074, China; 3 Hubei Key Laboratory of Marine Electromagnetic Detection and Control, Wuhan 430064, China; 4 Wuhan Second Ship Design and Research Institute, Wuhan 430064, China

**Keywords:** shaft-rate magnetic field detection, differential high-order dual-coupled Duffing oscillator, scale-dispersion joint statistical feature quantity, refined composite multiscale dispersion entropy

## Abstract

To address the issues of low signal-to-noise ratios in detecting weak shaft-rate magnetic fields of underwater targets in complex marine environments, the poor identification capability of traditional detection methods, and difficulty in recognizing unknown frequency signals, this study proposes a differential high-order dual-coupled Duffing oscillator detection method. A dual-oscillator model containing a cubic coupling term was constructed, and common-mode interference was suppressed through a differential structure. Simultaneously, a scale-dispersion joint statistical feature quantity was designed to automatically determine the critical threshold of system phase transition. Combined with refined composite multiscale dispersion entropy to quantitatively determine intermittent chaotic states, the method enabled the detection of weak shaft-rate magnetic fields with unknown frequencies and high-precision frequency estimation. Both simulation and marine experimental results demonstrate that compared with traditional Duffing oscillator detection methods, this approach significantly improved detection accuracy and reduced false alarm rates, making it applicable to underwater target monitoring, port security, and related scenarios.

**Dataset License:** license under which the dataset is made available (CC0, CC-BY, CC-BY-SA, CC-BY-NC, etc.)

## 1. Introduction

With the development of degaussing technology [1,2], the magnetic anomaly signals of underwater targets have been significantly weakened. Detecting them is extremely challenging using methods such as the orthogonal basis function [3,4,5,6], high-order cross [7], entropy filter [8], stochastic resonance [9], low-rank matrix decomposition [10], and deep learning [11,12,13]. In recent years, the shaft-rate magnetic field of underwater targets has attracted considerable attention. It originates from the modulation effect of corrosion-related currents [14] and is characterized by slow attenuation with distance and distinct line spectral features. Research on shaft-rate magnetic field detection technology will help improve the detection range and recognition accuracy of underwater targets.

Various solutions have been proposed to address the detection bottleneck of weak shaft-rate magnetic fields under complex magnetic interference. Hu et al. [15] studied a shaft-rate magnetic field detection method combining variational mode decomposition (VMD) and residual neural networks; however, the samples and model parameters relied on artificial priors. Lu et al. [16] applied a combined algorithm to shaft-rate magnetic field detection. This algorithm integrates successive VMD, Wasserstein distance-spectral entropy joint classification, enhanced spectral subtraction, and improved wavelet threshold denoising for multistage noise reduction. However, detection sensitivity is limited by the screening strategy of intrinsic mode functions. Li et al. [17] utilized a method based on the Rao detector and dynamic sliding thresholds to detect shaft-rate magnetic fields. This method adaptively sets decision thresholds using the sliding average of detection statistics, but the detection sensitivity depends on the accuracy of the noise parameter estimation. Wang et al. [18] employed an adaptive line spectrum enhancement method for shaft-rate magnetic field detection; however, it cannot suppress line spectrum interference in complex detection environments. Han et al. [19] designed a deep learning algorithm for shaft-rate magnetic field detection. This algorithm extracts time-domain envelope features and frequency-domain harmonic features of shaft-rate magnetic field signals through a dual-branch structure and uses interactive convolution to achieve cross-fusion of the two feature types. Nevertheless, the detection performance relies on the training datasets. Qiu et al. investigated two shaft-rate magnetic field detection methods: one combining the coherent signal enhancement method (CSEM), whitening filter (WF), VMD, and Duffing oscillator [20], while the other integrates robust CSEM with enhanced WF [21]. Both methods require dual-sensor synchronous measurements.

A detection method based on Duffing oscillators has become a powerful technique for weak signal detection under low signal-to-noise ratio (SNR) conditions, owing to its high sensitivity to weak periodic signals and insensitivity to noise. It has demonstrated excellent weak-signal-extraction performance in fields such as underwater acoustics [22,23], electroencephalography [24], and magnetic field [25,26] signal detection. Compared with the aforementioned detection methods, it utilizes the phase transition sensitivity of nonlinear dynamic systems to narrowband frequencies to capture weak periodic signals. Under low-SNR conditions, it requires no preprocessing or large training datasets and can achieve signal detection with a single sensor. However, traditional Duffing oscillator-based detection methods lack intuitiveness in determining results.

In addition, traditional detection methods based on the Duffing oscillator still have deficiencies in threshold determination, frequency estimation, and noise resistance improvement. In terms of threshold determination, the Poincaré section method [23] suffers from strong subjectivity and is not suitable for automatic computer identification, while the 0–1 test method [27] delivers poor calculation accuracy. For frequency estimation, the Duffing oscillator array method [28] involves heavy computational costs, and its frequency resolution is limited by the array step size, while the variable scale method [29] exhibits strong dependence on the scale transformation coefficient. With respect to noise resistance improvement, the method can effectively suppress white noise [30,31], yet the detection performance degrades in the presence of colored noise, yielding inferior noise resistance capability compared with the white noise background.

To enhance the recognition of detection results, strengthen engineering applicability, and further improve the capability to detect weak signals, this study proposes an underwater target shaft-rate magnetic field detection method based on differential high-order dual-coupled Duffing oscillators. The determination of the transition threshold of a chaotic system is key to accurately detecting weak signals using this Duffing oscillator system. A method for determining the transition threshold of the Duffing oscillator system based on the joint statistical characteristics of scale and dispersion is presented. Although the Duffing oscillator system can achieve weak signal detection under low-SNR conditions when the frequency of the signal to be measured is known, in practical applications, the frequency parameters of the signals to be detected are mostly unknown, which limits the application of this method to actual weak signal detection. To address this issue, this study applies the intermittent chaos method for shaft-rate magnetic field detection and proposes an intermittent chaos state determination method based on refined composite multiscale dispersion entropy, enabling weak signal detection with unknown frequencies under low SNR. Additionally, a calculation method for the frequency of the signal to be detected is provided.

## 2. Basic Theory

According to the generation mechanism, shaft-rate magnetic fields produced by shaft modulation can be equivalently modeled as a line current source with a fixed length. When the distance from the observation point to the center of the line current source exceeds three times the line current’s length, the magnetic field at that point can be approximated as a dipole field. Therefore, the shaft-rate magnetic field generated by underwater targets can be equivalently modeled as that produced by a time-harmonic horizontal electric dipole [32,33].

**Figure 1:**
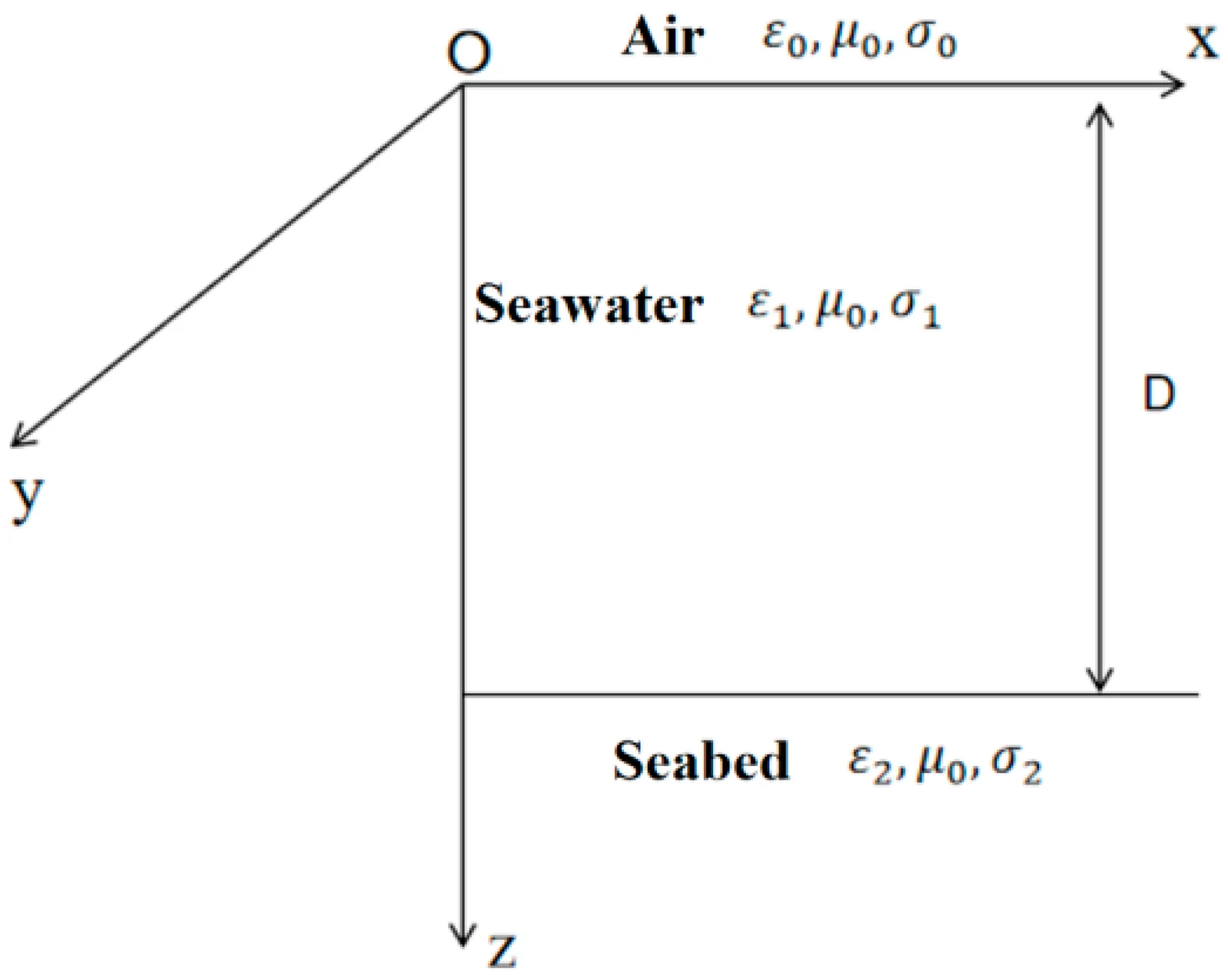
Schematic diagram of the air–seawater–seabed three-layer model.

In practical application scenarios, the presence of the seabed has a non-negligible impact on the propagation and distribution characteristics of electromagnetic fields in marine environments. To analyze the propagation laws of electromagnetic fields in these environments, it is necessary to establish a theoretical model of electromagnetic field propagation in a horizontally stratified marine environment consisting of air–seawater–seabed layers. This model makes the following assumptions about the marine environment: the overall environment consists of three layers of media, namely, air, seawater, and seabed, and each layer satisfies the properties of linearity, homogeneity, and isotropic media. Additionally, the model’s coordinate system is defined as follows: the *xy*-plane coincides entirely with the interface between the air and seawater, while the *z*-axis is oriented vertically downward and perpendicular to this interface, as shown in Figure 1.

The air–seawater interface is taken as the plane of *z* = 0, and the seawater–seabed interface as *z* = *D*. The air occupies the upper-half space of *z* < 0, the seawater occupies the 0 < *z* < *D* space, and the seabed occupies the *z* > *D* space. The electromagnetic parameters of air, seawater, and seabed are listed in Table 1.

**Figure 2:**
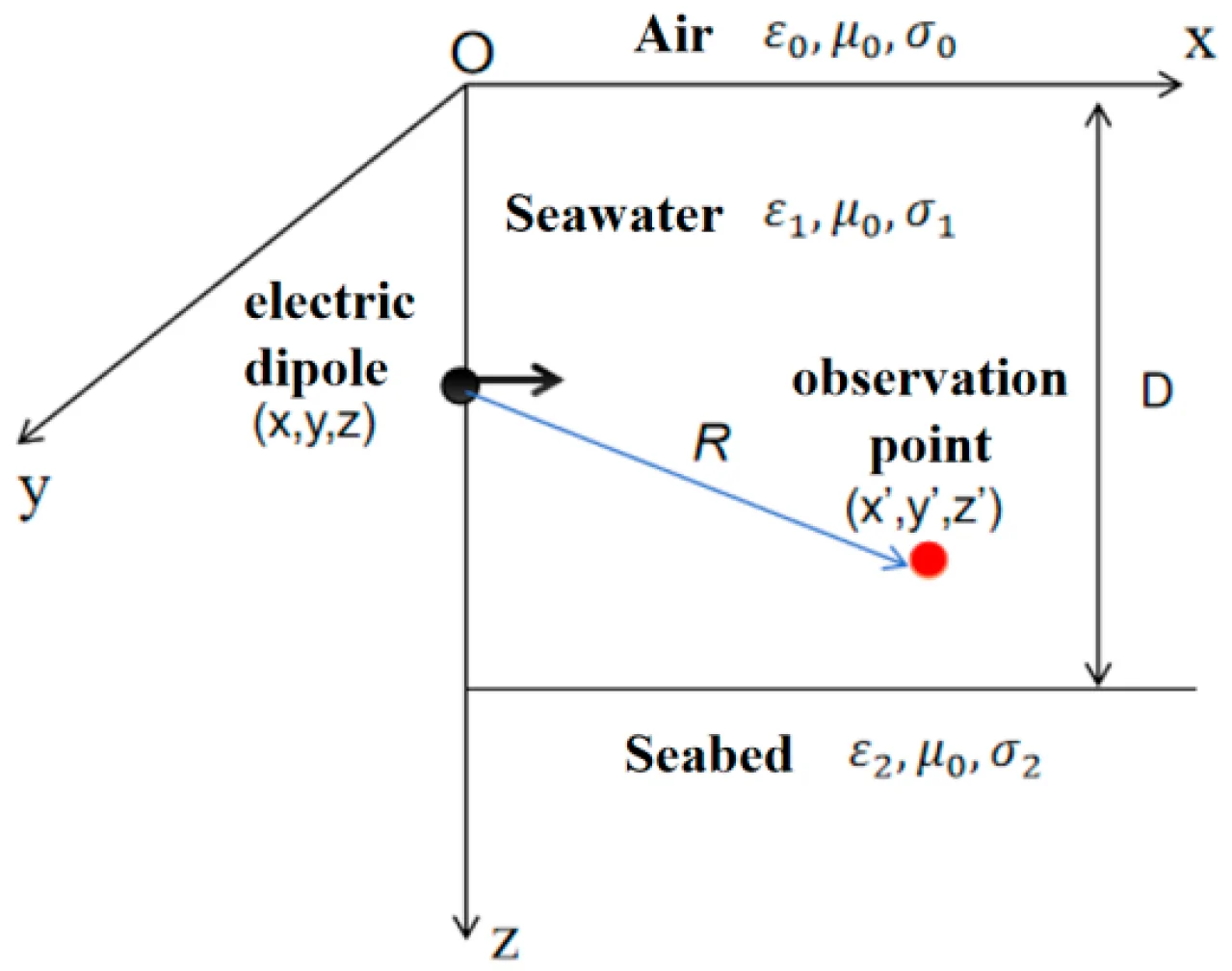
Alternating horizontal electric dipole in the three-layer model.

The time-harmonic dipole is distributed along the positive *x*-axis in seawater, with coordinates (*x*,*y*,*z*), and the observation point coordinates are (*x*’,*y*’,*z*’). The model is shown in Figure 2.

To satisfy the electromagnetic field boundary continuity conditions of the layered medium, the vector magnetic potential generated by the horizontal electric dipole can be decomposed into two parts. One is the tangential component in the same direction as the electric dipole moment, and the other is the normal component perpendicular to the medium boundary surface, i.e., $A_{n}\;=\;iA_{\mathrm{nx}}\;+\;kA_{\mathrm{nz}}$. For the air–seawater–seabed three-layer medium model, the vector magnetic potentials in the air, seawater, and seabed layers satisfy the corresponding constraint equation systems (1).

(1) $$\left\{\begin{array}{l} \nabla^{2}A_{0}=0\;(z\;<\;0)\\ \nabla^{2}A_{1}+{k_{1}}^{2}A_{1}=-\mu_{0}\mathrm{Ids}\delta (r)\;(0\;<\;z\;<\;D)\\ \nabla^{2}A_{2\;}+{{\;k}_{2}}^{2}A_{2}=0\;(z\;>\;D) \end{array}\right.$$

Here, $A_{0}$ represents the vector magnetic potential in the air layer, $A_{1}$ is the vector magnetic potential in the seawater layer, $A_{2}$ is the vector magnetic potential in the seabed layer, and Ids is the current element. δ(r) is the three-dimensional Dirac delta function, while $k_{1}$ and $k_{2}$ represent the complex wavenumbers of seawater and seabed seawater, respectively. The electromagnetic field boundary conditions for this model require that the tangential components of the electric and magnetic fields remain continuous at the medium interface. Let vector k be a normal vector perpendicular to the boundary surface. Then, k · H,k × H,and k × E satisfy continuity across the interface, and the corresponding mathematical expression is

(2) $$\left\{\begin{array}{l} k\;\cdot \nabla \times \;(A_{n}-A_{n+1})=0\\ k\;\times \nabla \times \;\left(A_{n}\;-\;A_{n+1}\right)=0\\ k\;\times \nabla \nabla \cdot \;(\frac{A_{n}}{\sigma_{n}}\;-\;\frac{A_{n+1}}{\sigma_{n+1}})=0 \end{array}\right.$$

By combining the above boundary conditions with the constraint equations of the vector magnetic potential, the solution is obtained. After deriving the expression for the vector magnetic potential A, applying partial differential operations yields the analytical expressions (3) for the three-component magnetic field in seawater.

(3) $$\left\{\begin{array}{c} B_{x}=-\frac{2(x-\;x^{\prime })(y\;-\;y^{\prime })}{r^{3}}\mathrm{MA}+\frac{\left(x-\;x^{\prime })(y-y^{\prime }\right)}{r^{2}}\mathrm{MB}\\ B_{y}=-\mathrm{MC}\left(z\;-\;z^{\prime }\right)+\mathrm{MD}\;-\;\left[\frac{1}{r}\;-\;\frac{{2\left(x\;-\;x^{\prime }\right)}^{2}}{r^{3}}\right]\mathrm{MA}\;-\frac{{\left(x\;-\;x^{\prime }\right)}^{2}}{r^{2}}\mathrm{MB}\\ B_{z}=\mathrm{MC}\left(y\;-\;y^{\prime }\right)+\frac{\left(y\;-\;y^{\prime }\right)}{r}\mathrm{ME} \end{array}\right.$$

where *M* represents the electric dipole moment, and *A*, *B*, *C*, *D*, and *E* can be expressed using Equation (4).

(4) $$\left\{\begin{array}{l} A\;=\int_{0}^{\infty }\left(f_{1z}e^{-u_{1}z}+g_{1z}e^{u_{1}z}\right)J_{1}\left(\lambda r\right)d\lambda \\ B\;=\int_{0}^{\infty }(f_{1z}e^{-u_{1}z}+g_{1z}e^{u_{1}z})\lambda J_{0}(\lambda r)d\lambda \\ \begin{array}{l} C\;=(\frac{1}{R^{3}}+\frac{ik_{1}}{R^{2}})e^{-ik_{1}R}\\ \begin{array}{l} D\;=\int_{0}^{\infty }(-f_{1x}e^{-u_{1}z}+g_{1x}e^{u_{1}z})J_{0}(\lambda r)u_{1}d\lambda \\ E\;=\int_{0}^{\infty }(f_{1x}e^{-u_{1}z}+g_{1x}e^{u_{1}z}){\lambda J}_{1}(\lambda r)d\lambda \end{array} \end{array} \end{array}\right.$$

Equations (3) and (4) provide rigorous mathematical analytical solutions for the low-frequency axial electromagnetic field in a marine environment based on the Sommerfeld integral. In these equations, r represents the radial projection distance in the horizontal direction, while R denotes the spatial straight-line distance from the time-harmonic dipole to the observation point, characterizing the magnetic field distribution pattern of a horizontal time-harmonic electric dipole in the three-layer air–seawater–seabed medium.

Assuming an electric dipole moment of 300 A·m, the frequency is 3 Hz and includes harmonics of 9 Hz and 15 Hz. The seawater depth is 200 m, with the observation point placed at a seabed depth of 195 m and a dipole depth of 80 m. According to the dipole spatial model, the dipole position coordinates are (0,0,80). The seawater conductivity is 3.7 S/m, the seabed conductivity is taken as 0.01 S/m, and the permeability is $\mu_{0}\;=\;4\pi \;\times {\;10}^{-7}\;H/m$.

**Figure 3:**
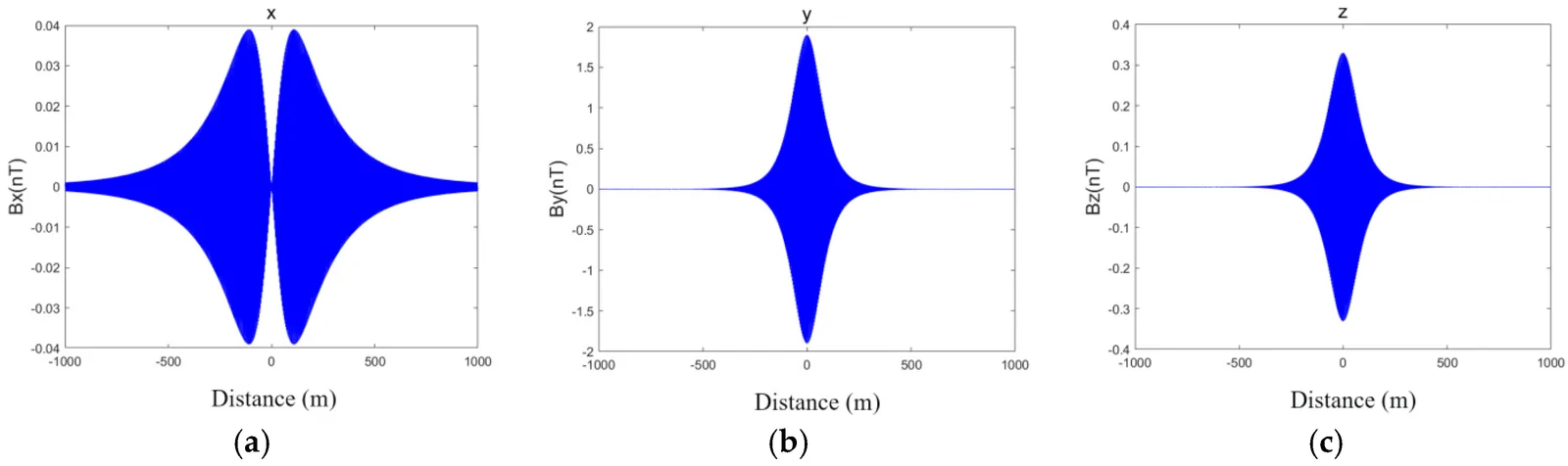
Three-axis magnetic field distribution of the horizontal time-harmonic dipole along the (*x*,20,195) survey line: (**a**) *x*-axis, (**b**) *y*-axis, and (**c**) *z*-axis component distributions.

Calculations were performed along the (*x*,20,195) survey line. When the x∈[−1000,1000] range varies, the changes in the three-axis magnetic field intensity are depicted in Figure 3.

**Figure 4:**
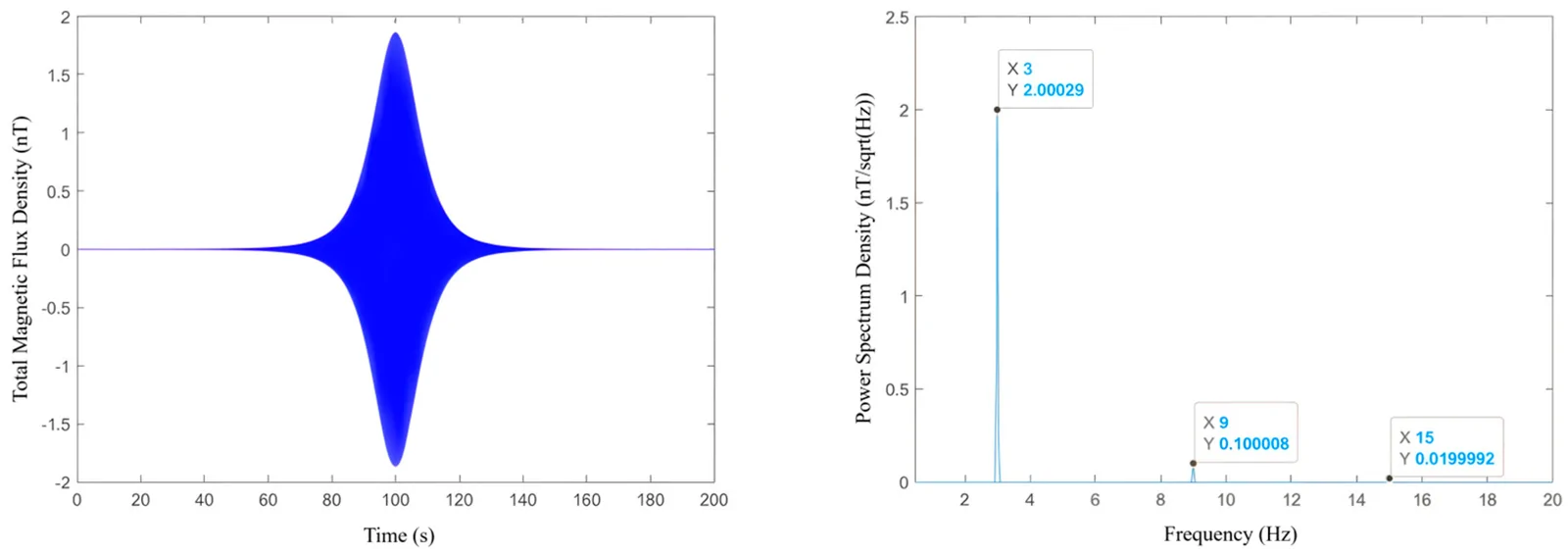
Total field and power density spectrum of the magnetic field generated by a horizontal time-harmonic dipole.

It can be observed that on the (*x*,20,195) survey line, the *B_x_* component exhibits two peaks, whereas the *B_y_* and *B_z_* components only show a single peak. When the observation point is directly below the magnetic dipole, the *B_x_* component has the smallest amplitude, almost approaching zero, whereas the *B_y_* and *B_z_* components reach their maximum magnetic field strength. Figure 4 illustrates the time-domain variation curve and corresponding spectrum of the total magnetic field when the observation point passes through a horizontal time-harmonic dipole along the (*x*,20,195) survey line.

From the time-varying curve of the total magnetic field of the horizontal time-harmonic dipole, the amplitude of the total magnetic field intensity reaches its maximum directly below the time-harmonic electric dipole, while the total field intensity monotonically attenuates with increasing distance from the dipole source. In the spectral analysis, distinct harmonics are present in addition to the fundamental frequency signal, which can serve as characteristic features of the shaft-rate magnetic field.

To demonstrate the validity of the electric dipole model, the simulated results were compared with measured shaft-rate magnetic field data reported by Zhang et al. [34] and Yue et al. [35].

It can be observed that the theoretically calculated curves are generally consistent with the measured curves in waveform structure, and their amplitudes are in good agreement, which verifies the correctness of the electric dipole model.

## 3. Methodology

### 3.1. Differential High-Order Dual-Coupled Duffing Oscillator Modeling Method

The Duffing oscillator is a typical nonlinear dynamic system, and its core advantages lie in its extreme sensitivity to periodic signals of the same frequency and insensitivity to Gaussian white noise. When the system operates at the critical point of chaos and a large-scale periodic phase transition occurs, tiny periodic signals with frequencies close to the internal driving frequency can trigger a state jump in the system, thereby enabling weak signal detection at low SNR. The basic Duffing oscillator equation [36] is

(5) $$\ddot{x}+k\dot{x}\;-\;\mathrm{ax}+bx^{3}=\gamma \cos\left(\omega t\right)$$

where x is the output of the Duffing oscillator system, k is the damping coefficient, $-\mathrm{ax}\;+\;bx^{3}$ is a nonlinear dynamic term, γ is the magnitude of the internal driving force, and ω is the internal driving frequency.

When used for weak axial frequency magnetic field signal detection, the Duffing oscillator equation must be updated to Equation (6).

(6) $$\ddot{x}+k\dot{x}\;-\;\mathrm{ax}+bx^{3}=\gamma \cos\left(\omega_{1}t\right)+A\cos\left(\omega_{2}t\right)+n\left(t\right)$$

Here, the damping coefficient, *k*, is set to 0.5, while the nonlinear dynamic term parameters, a and b, are both set to 1. A is the amplitude of the input signal, $\omega_{1}$ is the internal driving frequency, $\omega_{2}$ is the frequency of the signal to be detected, and *n*(*t*) is the noise.

The dual-coupled Duffing oscillator is a type of nonlinear dynamic system that exhibits unique response characteristics to external excitation. In this study, two sets of Duffing oscillators are subjected to high-order coupling, which enhances the new model’s sensitivity to periodic signals and improves its noise suppression capability. Additionally, by utilizing the phase difference between the outputs of the two coupled Duffing oscillators, common-mode interference can be eliminated. The newly established Duffing oscillator model can be described by Equation (7):

(7) $$\left\{\begin{array}{l} {\ddot{x}}_{1}+\;k{\dot{x}}_{1}\;-\;ax_{1}+\;b{x_{1}}^{3}+d{\left(y_{1}-\;x_{1}\right)}^{3}=\;\gamma \cos\left(\omega_{1}t\right)+A\cos\left(\omega_{2}t\right)+n\left(t\right)\\ {\ddot{y}}_{1}+\;k{\dot{y}}_{1}-\;ay_{1}+b{y_{1}}^{3}+d{\left(x_{1}-\;y_{1}\right)}^{3}=\;\alpha \gamma \cos\left(\omega_{1}t\right)+A\cos\left(\omega_{2}t\right)+n\left(t\right)\\ \begin{array}{c} {\ddot{x}}_{2}+\;k{\dot{x}}_{2}-\;ax_{2}+b{x_{2}}^{3}+d{\left(y_{2}-\;x_{2}\right)}^{3}\;=\;\gamma \cos\left(\omega_{1}t)+A\cos\left(\omega_{2}t\right)+n(t\right)\\ {\ddot{y}}_{2}+\;k{\dot{y}}_{2}-\;ay_{2}+b{y_{2}}^{3}+d{\left(x_{2}\;-\;y_{2}\right)}^{3}\;=\;\alpha \gamma \cos\left(\omega_{1}t\right)+A\cos\left(\omega_{2}t\right)+n(t) \end{array} \end{array}\right.$$

where the damping coefficient, *k*, and the high-order coupling coefficient, *d*, are both set to 0.5, while the parameters of the nonlinear dynamic terms, a and b, are both set to 1. α is used to alter the phase of the two oscillator groups, which affects the common-mode interference suppression effect, discernibility of chaotic states, and detection threshold, with a value of 1.001. $x_{i}$ and $y_{i}$ are the state variables of the system, and ${\dot{x}}_{i}$, ${\dot{y}}_{i}$, ${\ddot{x}}_{i}$, and ${\ddot{y}}_{i}$ represent the first and second time derivatives of $x_{i}$ and $y_{i}$, respectively.

This study adopts a third-order nonlinear coupling term, *d*(*y*−*x*)^3^, to realize mutual constraints between two oscillators. Different from linear coupling, the constraint strength of third-order coupling varies rapidly with the displacement difference between two oscillators. When the oscillators behave similarly with a small displacement difference, the mutual constraint induced by coupling is weak; when obvious displacement deviation occurs, the coupling restraint increases rapidly and imposes strong nonlinear restrictions on the dynamic evolution of both oscillators. The third-order coupling modifies the bifurcation condition of the whole coupled system and reshapes the phase trajectory topological structure of chaotic attractors and homoclinic orbits. Under appropriate coupling strength, third-order coupling will not eliminate chaos but adjusts the critical threshold for transition between the chaotic state and large-scale periodic state. The small disturbance caused by noise only blurs the boundary of phase trajectories and cannot trigger state transitions. By contrast, a weak periodic target signal can break critical equilibrium and drive the system into large-scale periodic motion, so as to improve sensitivity to periodic signals and enhance noise robustness.

If *d* is excessively large, the amplification effect of *d*(*y*−*x*)^3^ produces a strong coupling constraint, which severely restricts the free evolution of two oscillators. The system can hardly stay at critical chaos, and a weak periodic signal cannot trigger state transitions, resulting in degradation of detection sensitivity. If *d* is too small, the nonlinear restraint of third-order coupling cannot be activated, and the two oscillators work almost independently, degenerating into two isolated Duffing oscillators without noise resistance improvement brought on by coupling.

Two groups of high-order dual-coupled Duffing oscillators receive identical measured signals and background noise. As common mode disturbance, the noise produces nearly synchronous motion offset in both oscillators. After the difference operation $x_{1}-x_{2}$, the synchronous common mode noise is counteracted. A tiny mismatch factor, α, is introduced for the internal driving force. The slight mismatch barely influences noise-induced motion yet produces a small phase difference between outputs of two coupled systems under a periodic signal. Such a phase-difference-carrying signal-induced state feature is preserved in the differential time series output.

As shown in Equation (8), the target signal is detected by performing differential calculations on the state solutions of the two high-order coupled Duffing oscillators, leveraging the differences between the large-scale periodic and chaotic states of the dual-coupled Duffing oscillators in the time domain. Unlike traditional phase diagram judgment methods, the differential high-order dual-coupled Duffing oscillator system employs differential time-series plots for determination, making the detection results more intuitive and easier to distinguish.

(8) $$x_{\mathrm{difference}}=\;x_{1}-x_{2}$$

**Figure 5:**
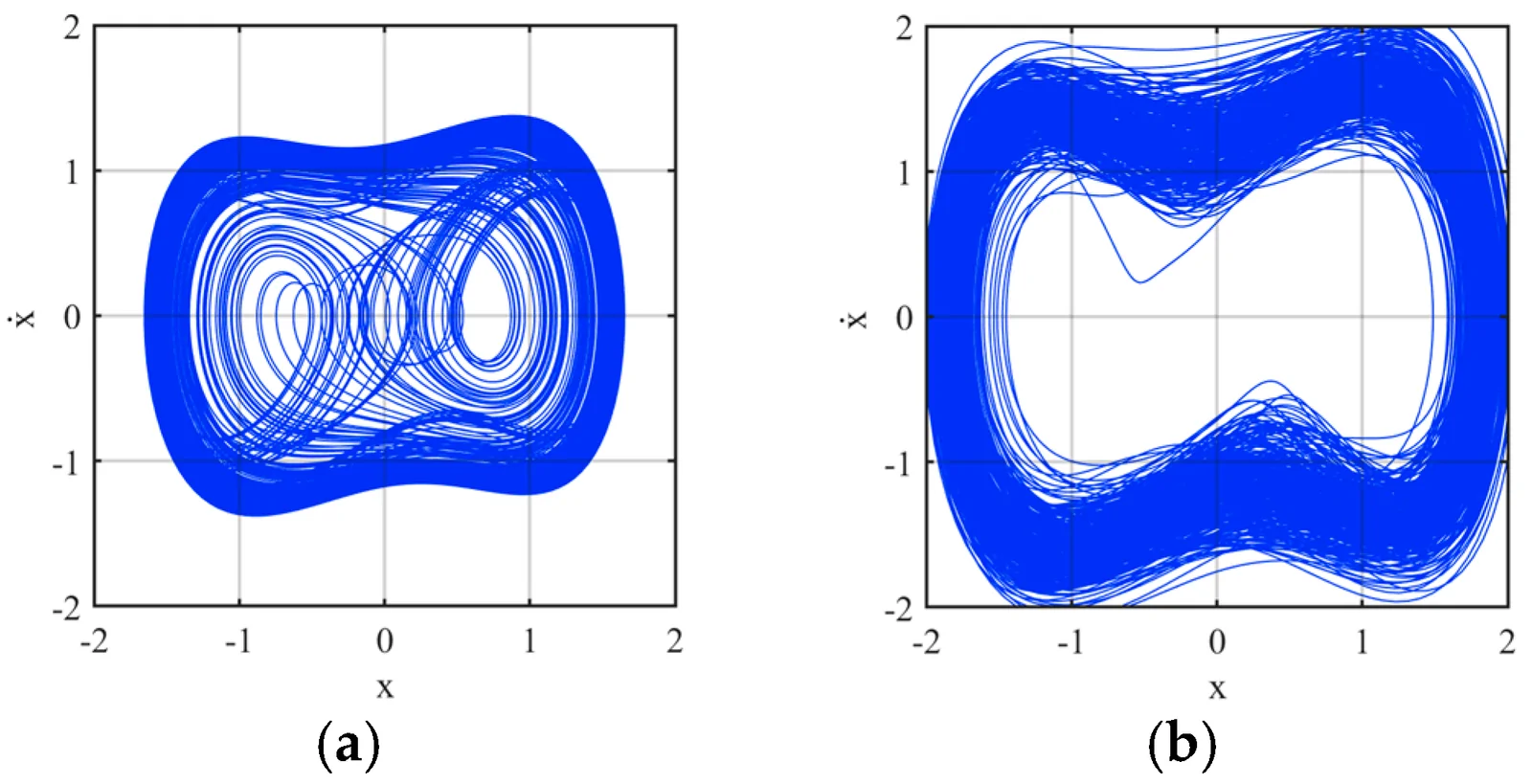
Comparison of phase diagrams for traditional Duffing oscillator: (**a**) chaotic state; (**b**) large-scale periodic state.

**Figure 6:**
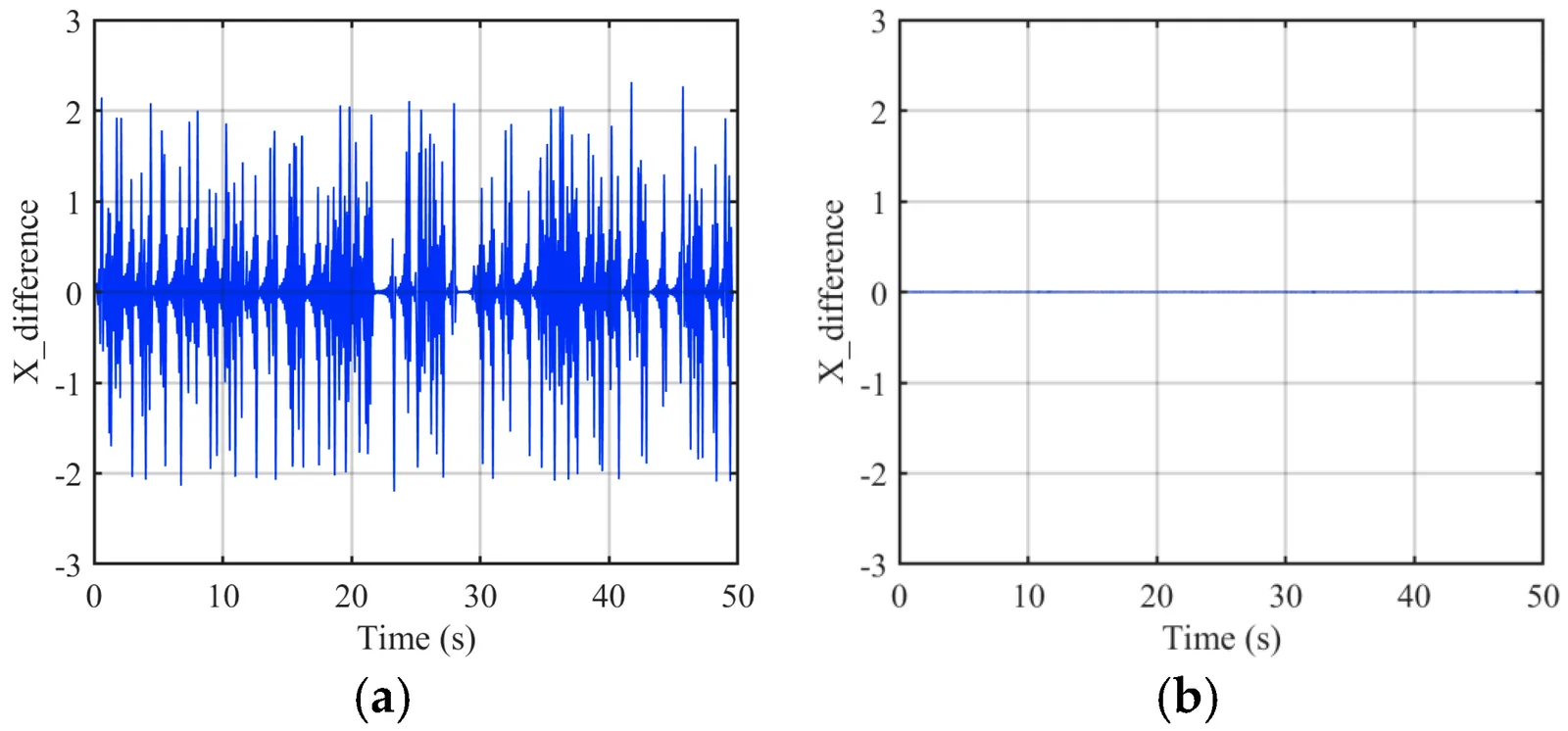
Comparison of time-series diagrams for differential high-order dual-coupled Duffing oscillator detection method: (**a**) chaotic state; (**b**) large-scale periodic state.

For a better illustration, a comparison is made between the differential high-order dual-coupled Duffing oscillator detection method and the traditional Duffing oscillator detection method. The parameters are set as follows: internal driving force magnitude, γ = 0.817; internal driving frequency, $\omega_{1}\;=\;44\;\mathrm{rad}/s$; input signal amplitude, A = 0.22, signal frequency to be detected $\omega_{2}\;=\;44\;\mathrm{rad}/s$; and Gaussian white noise intensity, D = 0.12. Before inputting the signal to be detected, the Duffing oscillator system is in a chaotic state. After inputting, the Duffing oscillator system transitions to a large-scale periodic state. As illustrated in Figure 5, if the traditional Duffing oscillator detection method is used, the phase diagram determination lacks intuitive clarity. In contrast, if the differential high-order dual-coupled Duffing oscillator detection method is employed (Figure 6), the chaotic and large-scale periodic states can be easily distinguished from the time-series diagram.

### 3.2. A Method for Determining the Threshold of the Internal Driving Force Based on Joint Statistical Characteristics of Scale and Dispersion

The selection of the critical threshold for internal driving force is a core prerequisite for the differential high-order dual-coupled Duffing oscillator to achieve weak periodic signal detection. Its essence lies in controlling the initial dynamic state of the chaotic system through the threshold, ensuring that weak target signals can trigger a significant phase transition from the chaotic state to the large-scale periodic state, thereby completing the identification of target signals. Weak target signals, such as shaft-rate magnetic fields, have low amplitudes and are highly susceptible to noise interference. The rationality of the threshold selection directly determines the system’s detection performance. If the threshold is extremely small, the system is prone to false phase transitions triggered by noise disturbances, leading to excessive false alarm rates. If the threshold is excessively large, the amplitude of the internal driving force already exceeds the critical phase transition point, causing the system to initially reside in a large-scale periodic state, thereby losing the ability to discern target signals through state transitions, ultimately resulting in missed detection. Therefore, the setting of the critical threshold for the internal driving force is a key step in applying differential high-order dual-coupled Duffing oscillators.

To address the aforementioned issue, this study proposes a quantitative method for determining the critical threshold of the internal driving force that balances accuracy and efficiency based on the fundamental laws of state transitions in chaotic systems. The evolution of the Duffing oscillator from a chaotic to a large-scale periodic state is essentially a transition of the system’s motion time series from disorder to order. The spatial distribution characteristics of phase trajectories can reflect this regularity of order-state changes.

For the differential high-order dual-coupled system, the output quantities of the two sets of coupled oscillators are first differentially processed to construct the system’s characteristic phase diagram. The differential result of the x values of the two sets of oscillators serves as the horizontal coordinate of the phase diagram, while that of the y values serves as the vertical coordinate of the phase diagram, as indicated in Equations (8) and (9).

(9) $$y_{\mathrm{difference}}=y_{1}-\;y_{2}$$

**Figure 7:**
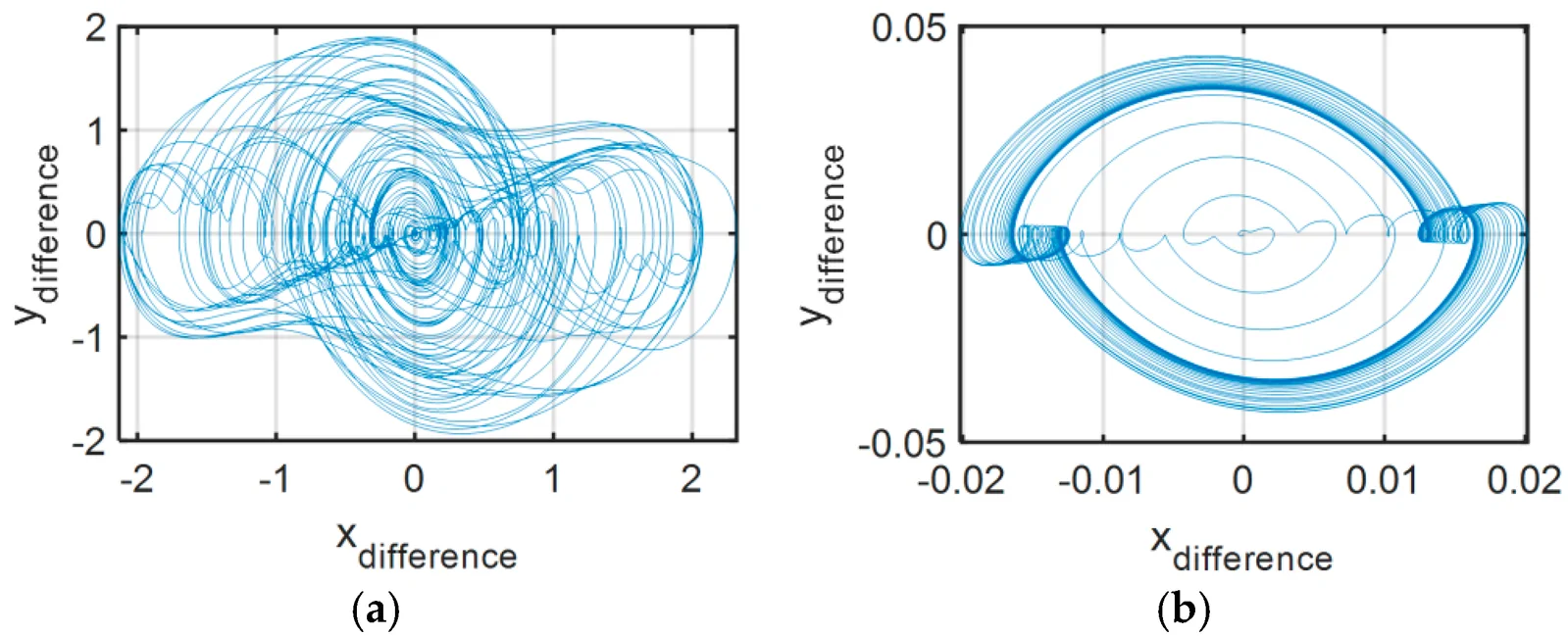
Phase diagrams of the two states of the differential high-order dual-coupled Duffing oscillator: (**a**) chaotic state; (**b**) periodic state.

For the differential high-order dual-coupled Duffing oscillator, the characteristic differences in phase trajectories between the chaotic and large-scale periodic states are evident, as illustrated in Figure 7.

Figure 7a displays the phase diagram of the chaotic state of the differential high-order dual-coupled Duffing oscillator. The phase trajectories of the chaotic state exhibit irregular, non-periodic winding and folding, with high spatial-distribution dispersion. Figure 7b shows that the phase trajectories of the large-scale periodic state converge into regular closed periodic orbits with a highly concentrated spatial distribution. The geometric morphological differences between the two states are significant, intuitively reflecting the evolution law of the system’s order state.

To quantitatively describe the geometric distribution characteristics of the phase trajectories of the differential high-order dual-coupled Duffing oscillator, this study defines a joint statistical feature quantity of scale and dispersion (SDJS) to achieve quantitative discrimination of system states. The phase trajectory point set of the differential high-order dual-coupled Duffing oscillator can be represented by Equation (10).

(10) $${\left\{\left(x_{i},y_{i}\right)\right\}}_{i=1}^{N}$$

Here, $x_{i}$ is the i-th point of $x_{\mathrm{difference}}$, $y_{i}$ is the i-th point of $y_{\mathrm{difference}}$, and *N* is the total number of points. The Euclidean distance from each point in the phase diagram to the origin can be expressed by Equation (11).

(11) $$r_{i}=\sqrt{\left(X_{i}^{2}+Y_{i}^{2}\right)}$$

Equation (12) defines the average value of the Euclidean distance.

(12) $$d=\frac{1}{N}\sum_{i=1}^{N}\sqrt{\left(X_{i}^{2}+Y_{i}^{2}\right)}$$

This average value reflects the average scale of the overall energy distribution of the phase trajectory. In chaotic states, the phase trajectories are more dispersed, resulting in larger average distances, whereas in periodic states, the trajectories concentrate on closed loops, leading to smaller average distances. However, using only the average distance is insufficient to distinguish between the two states, as they may have similar average radii but exhibit significant differences in distribution dispersion. The standard deviation is a statistical measure that reflects the dispersion of data. In probability and statistics, it is commonly used to quantify the dispersion of statistical distributions. Therefore, the standard deviations of the x- and y-differential output values are introduced into the feature quantity, as shown in Equation (13).

(13) $$\left\{\begin{array}{c} \sigma_{x}=\frac{1}{N}\sqrt{{\left(x_{i}\;-\;\overset{-}{x}\right)}^{2}}\\ \sigma_{y}=\frac{1}{N}\sqrt{{\left(y_{i}\;-\;\overset{-}{y}\right)}^{2}} \end{array}\right.$$

The average Euclidean distance *d* characterizes the global average spatial scale of the phase trajectory, while the standard deviations $\sigma_{x}$ and $\sigma_{y}$ jointly quantify the degree of distribution dispersion of the phase diagram along each coordinate axis. Considering both the spatial scale and distribution dispersion characteristics of the phase diagram, a joint feature quantity is constructed, as shown in Equation (14).

(14) $$\mathrm{SDJS}=\sigma_{x}\;\times {\;\sigma }_{y}\;\times \;d$$

Following the above steps, the results in Figure 7 are analyzed. When the differential high-order dual-coupled Duffing oscillator is in a chaotic state, its *SDJS* characteristic value is 0.36. However, when it is in a large-scale periodic state, its *SDJS* value is $3.71\;\times \;10^{-6}$. The results indicate that the *SDJS* characteristic value of the chaotic state significantly differs from that of the large-scale periodic state. This demonstrates that when the differential high-order dual-coupled Duffing oscillator transitions from a chaotic state to a large-scale periodic state, *SDJS* undergoes a noticeable change, which can serve as an effective method for determining the optimal threshold.

**Figure 8:**
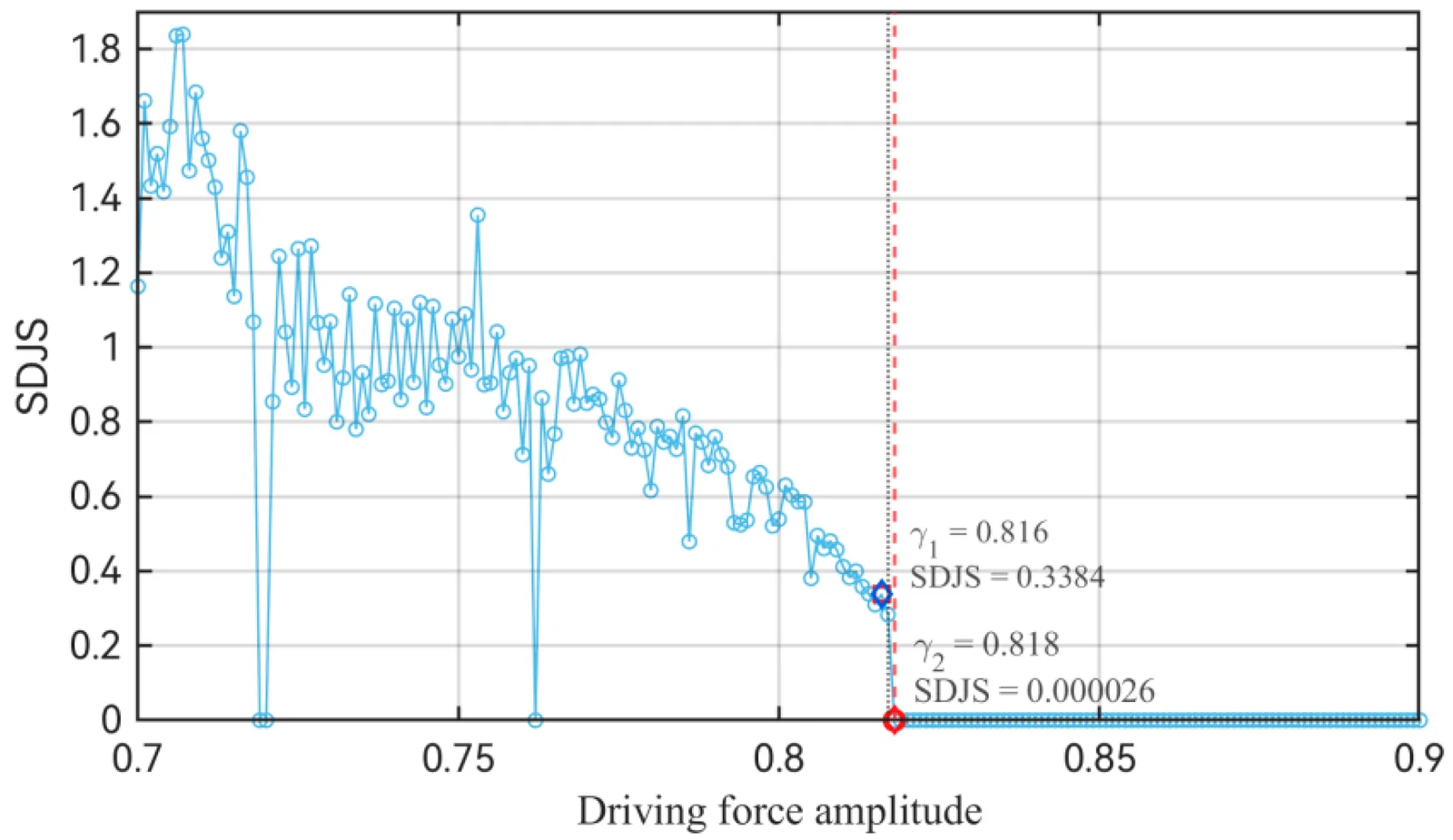
*SDJS* eigenvalue variation with internal driving force amplitude.

In the simulation of the differential high-order dual-coupled Duffing oscillator, the internal driving frequency, $\omega_{1}$, is set to 1 Hz, the sampling rate to 1000 Hz, and the sampling duration to 50 s. The coupling coefficient, *d*, is set to 0.5, and the two characteristic parameters of the nonlinear dynamic term, *a* and *b*, are both set to 1. The parameter α is set to 1.001 and is used to adjust the phase difference between the two sets of Duffing oscillators in the dual-coupled system. In the absence of an external signal input, the internal driving force magnitude, *γ*, is adjusted within the range of 0.7–0.9. To capture the critical characteristics of the system’s phase transition, the adjustment step size is set to 0.001, and the evolution of the system’s motion state is analyzed by gradually increasing *γ*. The *SDJS* eigenvalue of the differential high-order dual-coupled Duffing oscillator as a function of the internal driving force amplitude is depicted in Figure 8.

Figure 8 shows that when the amplitude of the internal driving force is 0.816, the *SDJS* eigenvalue is 0.3384. As the amplitude of the internal driving force increases and reaches 0.818, the *SDJS* eigenvalue becomes 2.6 × 10^−5^. At this point, the eigenvalue drops sharply and remains at an extremely low level, indicating that the system transitions from a chaotic to a large-scale periodic state. From Figure 8, it is evident that when the amplitude of the internal driving force is 0.817, the system is in a critical state. Therefore, 0.817 can be considered as the critical threshold for the internal driving force of the differential high-order dual-coupled Duffing oscillator. The *SDJS* feature quantity has low computational complexity, requiring only a single traversal for completion, making it suitable for real-time processing.

### 3.3. Signal Detection Method Based on Intermittent Chaos

When the Duffing oscillator is used for weak signal detection, if the measured value contains a target signal with the same frequency as the internal driving force, the system will exhibit a significant phase transition. For the traditional single Duffing oscillator system, the system transitions from a chaotic to a large-scale periodic state, and the corresponding entropy value decreases. For the differential high-order dual-coupled Duffing oscillator, the time-domain waveform of the system output changes from irregular oscillations to small-amplitude steady fluctuations. The traditional Duffing oscillator can only effectively detect signals with known frequencies. To achieve effective detection of shaft-rate magnetic field signals with unknown frequencies, this study proposes a differential high-order dual-coupled Duffing oscillator system based on intermittent chaos.

**Figure 9:**
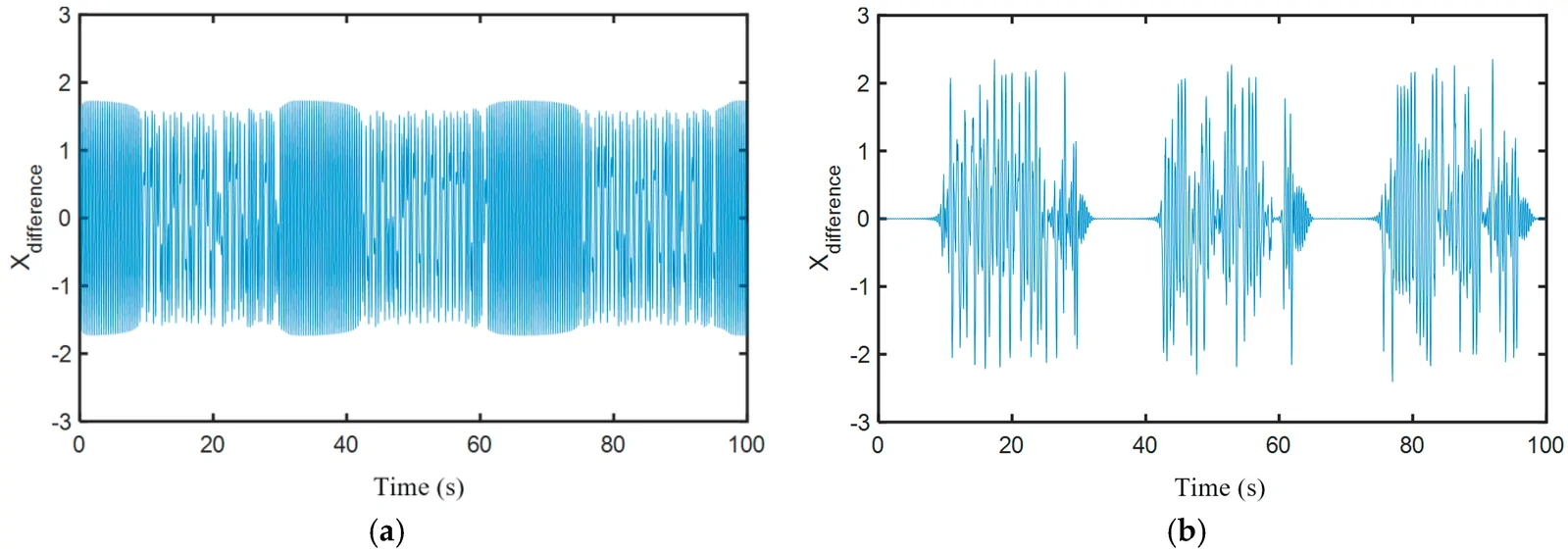
Comparison of intermittent chaotic states between (**a**) single Duffing oscillator and (**b**) differential high-order dual-coupled Duffing oscillator.

When there is a frequency difference, ∆ω, between the target signal and internal driving force, the system exhibits periodic changes, as depicted in Figure 9. At this time, the system alternates between chaotic and large-scale periodic states, a phenomenon known as intermittent chaos, with a variation period of T = 2π∆ω [37]. Figure 9b shows that for the differential high-order dual-coupled Duffing oscillator system, the large-scale periodic state is significantly suppressed, while the chaotic state is amplified, making the intermittent chaotic state highly pronounced.

The frequency-difference condition for the occurrence of intermittent chaotic states in the differential high-order dual-coupled Duffing oscillator is not arbitrary; it must satisfy the inherent constraints of the system’s dynamic response. When Δω exceeds a certain critical value, the hysteresis in the system’s dynamic response will prevent it from promptly following the rapid changes in the equivalent driving force, thereby suppressing the intermittent chaotic characteristics and rendering the system unable to effectively identify the target signal.

As reported in [22], when $\left|\Delta \omega /\omega \right|\;\leq \;0.09$ is satisfied, the system can generate intermittent chaotic states. The reference frequency, $\omega_{0}$, can be set to 1 rad/s, and the internal driving frequency is incrementally swept according to the rule of $\omega_{n}\;=\;1.05^{n}\omega_{0}$, while the time-domain output characteristics of the system at each sweep point are observed in real time. Intermittent chaotic states typically occur near $\omega_{n}$ and $\omega_{n+1}$, which are close to the frequency to be detected, while other frequencies remain in a chaotic state. Based on this characteristic, it can be determined that the frequency of the measured shaft signal falls between $\omega_{n}$ and $\omega_{n+1}$, and the average of these two values can be used for representation.

However, the existing state determination methods for Duffing oscillator systems often fail to meet application requirements due to the following reasons: First, the intuitive phase diagram determination method relies heavily on subjective human experience. Under strong noise interference, the chaotic and large-scale periodic characteristics of phase trajectories are easily blurred, leading to frequent misjudgments. Second, traditional quantitative determination methods, such as Lyapunov exponents, fractal dimensions, and multiscale sample entropy, suffer from high computational complexity and poor real-time performance, severely limiting their applications.

Entropy quantifies the degree of disorder and complexity variations of a system [38]. It is capable of identifying abrupt dynamic transitions when intermittent chaos switches back and forth between regular and chaotic motions, thus enabling the determination of intermittent chaotic states. Refined composite multiscale dispersion entropy (RCMDE) [39] is a powerful tool for analyzing signals, owing to its outstanding stability, comprehensive information capture, and computational efficiency. It can effectively characterize the complexity of dynamic behaviors in chaotic systems and exhibits strong determination capabilities for intermittent chaotic states. The differential high-order dual-coupled Duffing oscillator displays distinct alternating periodic and chaotic features during intermittent chaos. This study utilizes *RCMDE* to determine the state results of the Duffing oscillator, and the computational process is described as follows.

First, set the embedding dimension to m = 2, category number to c = 6, time delay to d = 1, and maximum scale factor to $\tau_{\max}\;=\;20$, and substitute them into the differential output signal of the differential high-order dual-coupled Duffing oscillator.

Then, for the differential output signal $u\;=\;\{u_{1},u_{2},\ldots ,u_{L}\}$, for each scale factor, τ, generate τ different coarse-grained time series, each with a different starting point. The k-th coarse-grained sequence is defined by Equation (15).

(15) $$x_{k,j}^{\left(\tau \right)}=\frac{1}{\tau }\sum_{b=k+\tau \left(j-1\right)}^{k+\tau j-1}u_{b},\;1\;\leq \;j\;\leq \;N,\;1\;\leq \;k\;\leq \;\tau$$

Here, *N* = [*L*/*τ*], indicating the floor function applied to L/τ.

Next, for each $x_{k}^{\left(\tau \right)}$, use the mean and standard deviation of the original signal to map each point to the [0, 1] interval via the normal cumulative distribution function. Then, linearly quantize the mapped values into c integer classes (from 1 to *c*) and construct embedding vectors with an embedding dimension of m and a time delay of *d*. Subsequently, count the occurrence frequency, $p_{k}^{\left(\tau \right)}\left(\pi \right)$, of all possible $c^{m}$ dispersion patterns.

Furthermore, for each dispersion pattern, π, calculate its average occurrence frequency across τ coarse-grained sequences using Equation (16).

(16) $$\overset{⃐}{p}\left(\pi \right)=\frac{1}{\tau }\sum_{k=1}^{\tau }p_{k}^{\left(\tau \right)}\left(\pi \right)$$

Finally, compute the entropy value by averaging the pattern probabilities using Equation (17).

(17) $$\mathrm{RCMDE}\left(x,m,c,d,\tau \right)\;=\;-\frac{\sum_{\pi =1}^{c^{m}}\overset{⃐}{p}\left(\pi \right)\cdot \ln\left(\overset{⃐}{p}\left(\pi \right)\right)}{\ln\left(c^{m}\right)}$$

*RCMDE* can effectively describe the complexity of time series at different time scales. Generally, the more complex the time series, the larger the *RCMDE* value. In actual complex marine detection environments, the entropy value output by the system is affected by environmental noise, leading to blurred boundaries in state determination. To overcome this limitation, this study introduces a dynamic threshold mechanism. It uses the *RCMDE* mean value under environmental noise input as the zero baseline, ${\mathrm{RCMDE}}_{\mathrm{base}}$, and utilizes ${\mathrm{RCMDE}}_{\mathrm{test}}$ during the input of the measured signal to construct a relative entropy change feature, $\Delta \mathrm{RCMDE}\;=\;{\mathrm{RCMDE}}_{\mathrm{base}}\;-\;{\mathrm{RCMDE}}_{\mathrm{test}}$. By setting the determination threshold using the *RCMDE* under environmental noise input, the target signal is considered detected when the relative entropy change, ΔRCMDE, output by the system exceeds this threshold.

## 4. Experiments and Analysis

### 4.1. Simulation Verification

To systematically verify the detection performance of the proposed method, a simulated dataset was constructed to mimic real marine detection scenarios. The simulated signal consisted of shaft-rate magnetic field signals superimposed with 1/f noise. The shaft-rate magnetic field signal was calculated based on a time-harmonic magnetic dipole model to simulate the shaft-rate magnetic field of a moving target. The core parameters are listed in Table 2.

**Figure 10:**
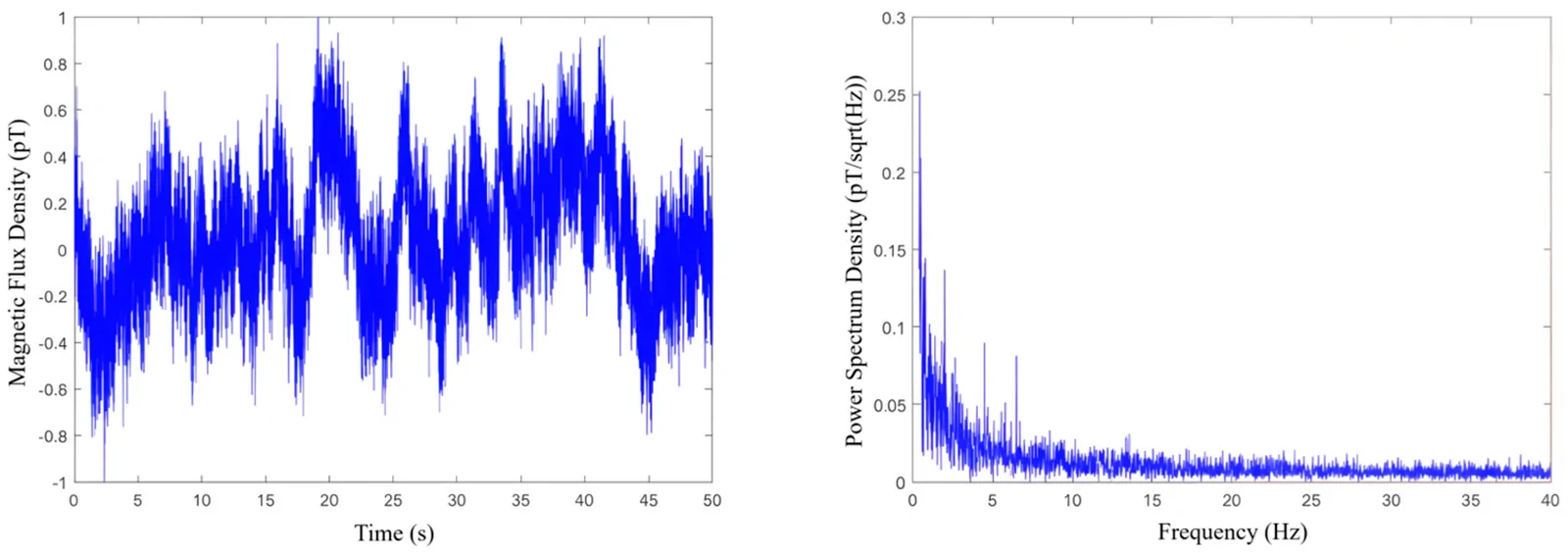
Shaft-rate magnetic field simulation signal under noise interference.

The simulated signal is shown in Figure 10. Based on the shaft-rate magnetic field signal, 1/f noise with an SNR of −25 dB was added. Owing to strong noise interference, the frequency of the target shaft-rate magnetic field signal could not be identified through frequency-domain or time-domain analysis.

**Figure 11:**
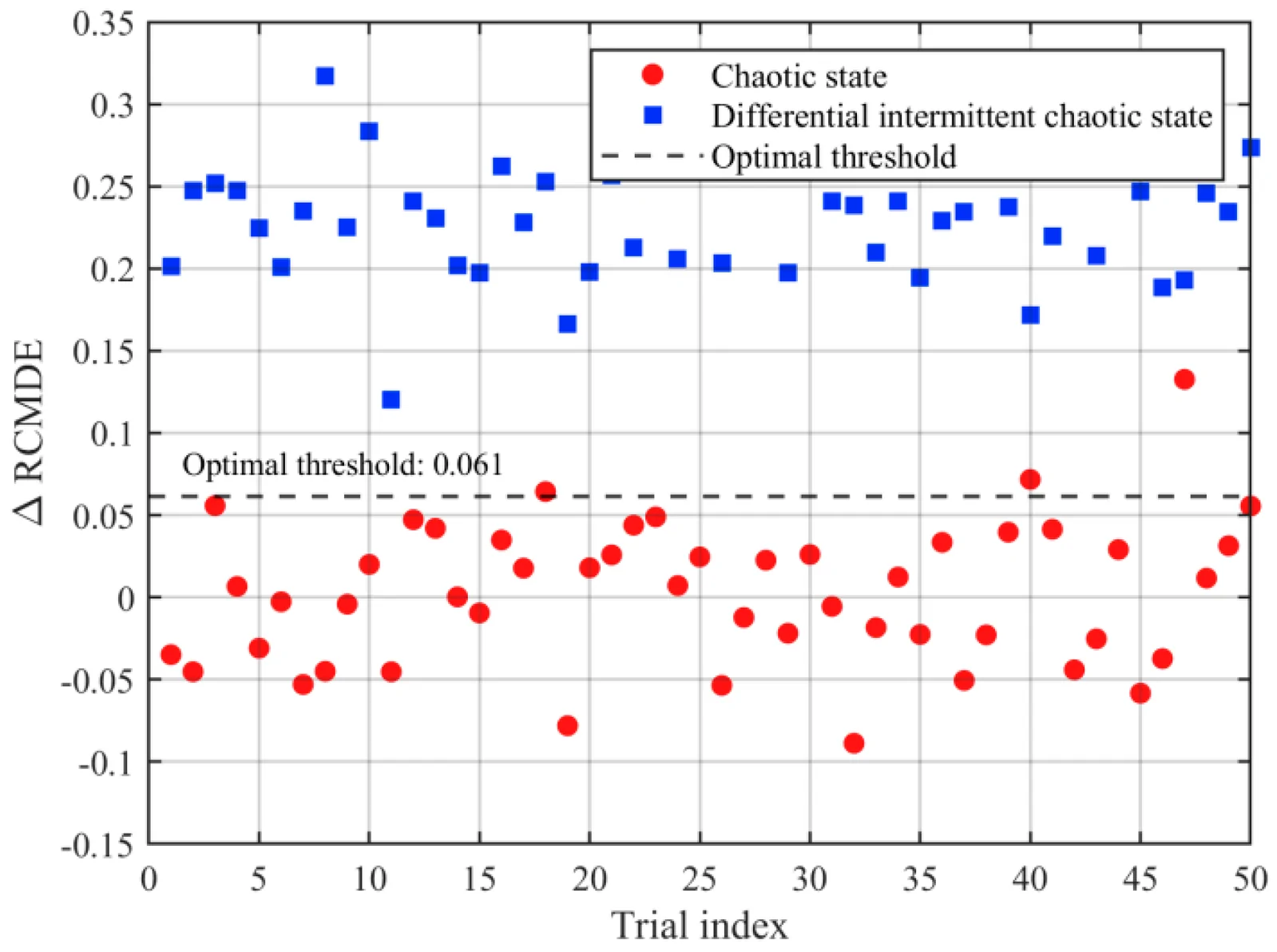
Relative entropy variation features under different states.

Subsequently, a differential high-order dual-coupled Duffing oscillator was utilized to detect the shaft-rate magnetic field signal. First, the judgment threshold for intermittent chaotic states was determined. Under the −25 dB low-SNR condition, Monte Carlo simulation experiments were conducted. The *RCMDE* values of 50 noise samples and 50 noisy signal samples were calculated. Then, using the mean *RCMDE* of the noise samples as the zero baseline (calculated as ${\mathrm{RCMDE}}_{\mathrm{base}}\;=\;0.85$), the relative entropy variation features, ΔRCMDE, of the aforementioned 100 samples were computed. Finally, from Figure 11, the optimal judgment threshold for distinguishing intermittent chaotic states was determined to be 0.061.

**Figure 12:**
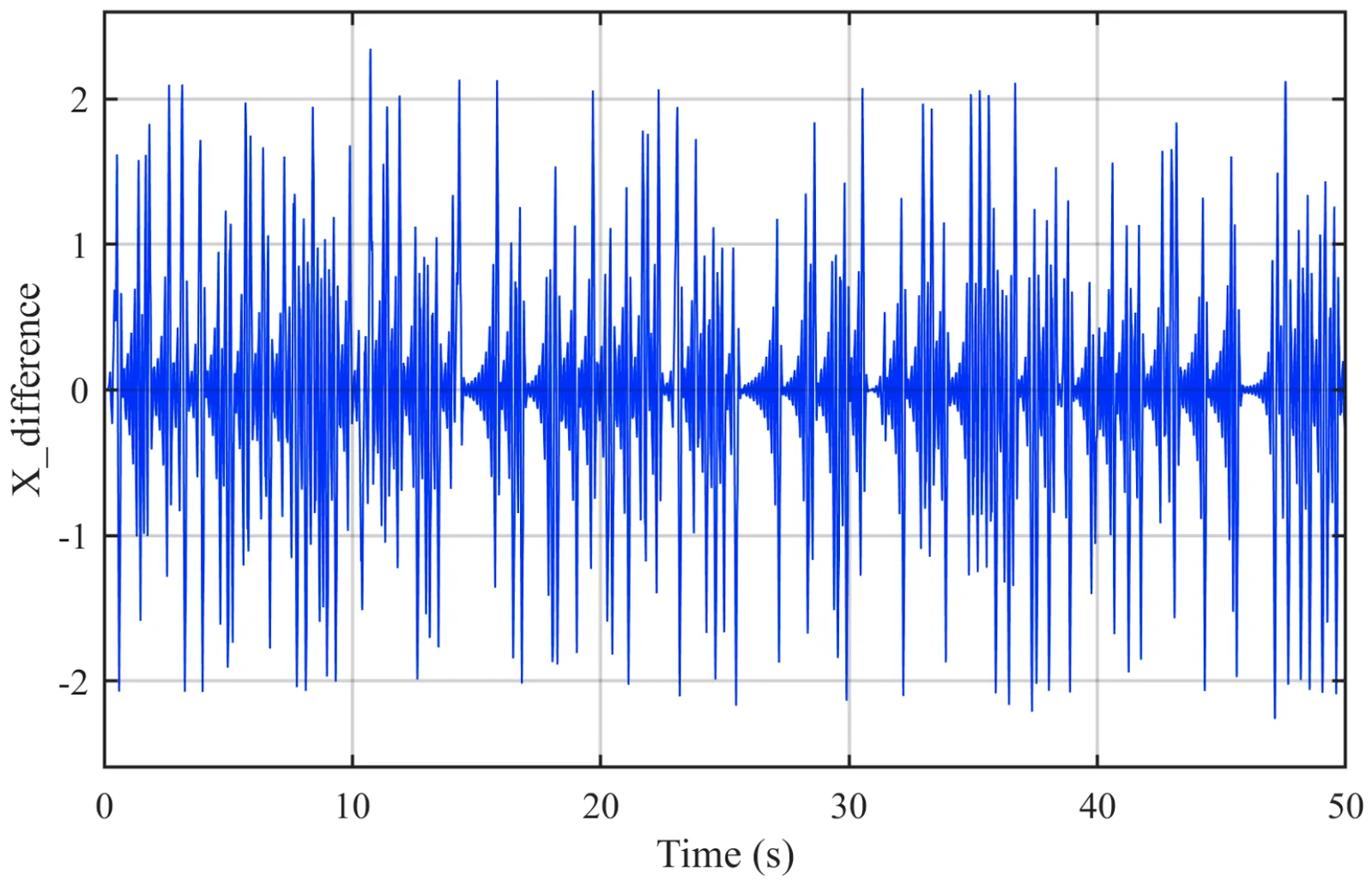
Differential output of the differential high-order dual-coupled Duffing oscillator with input noise.

For the differential high-order dual-coupled Duffing oscillator system, when noise was input, the differential output of the Duffing oscillator was as shown in Figure 12. Because the system amplified the chaotic states, the output results exhibited a significantly disordered and chaotic state.

**Figure 13:**
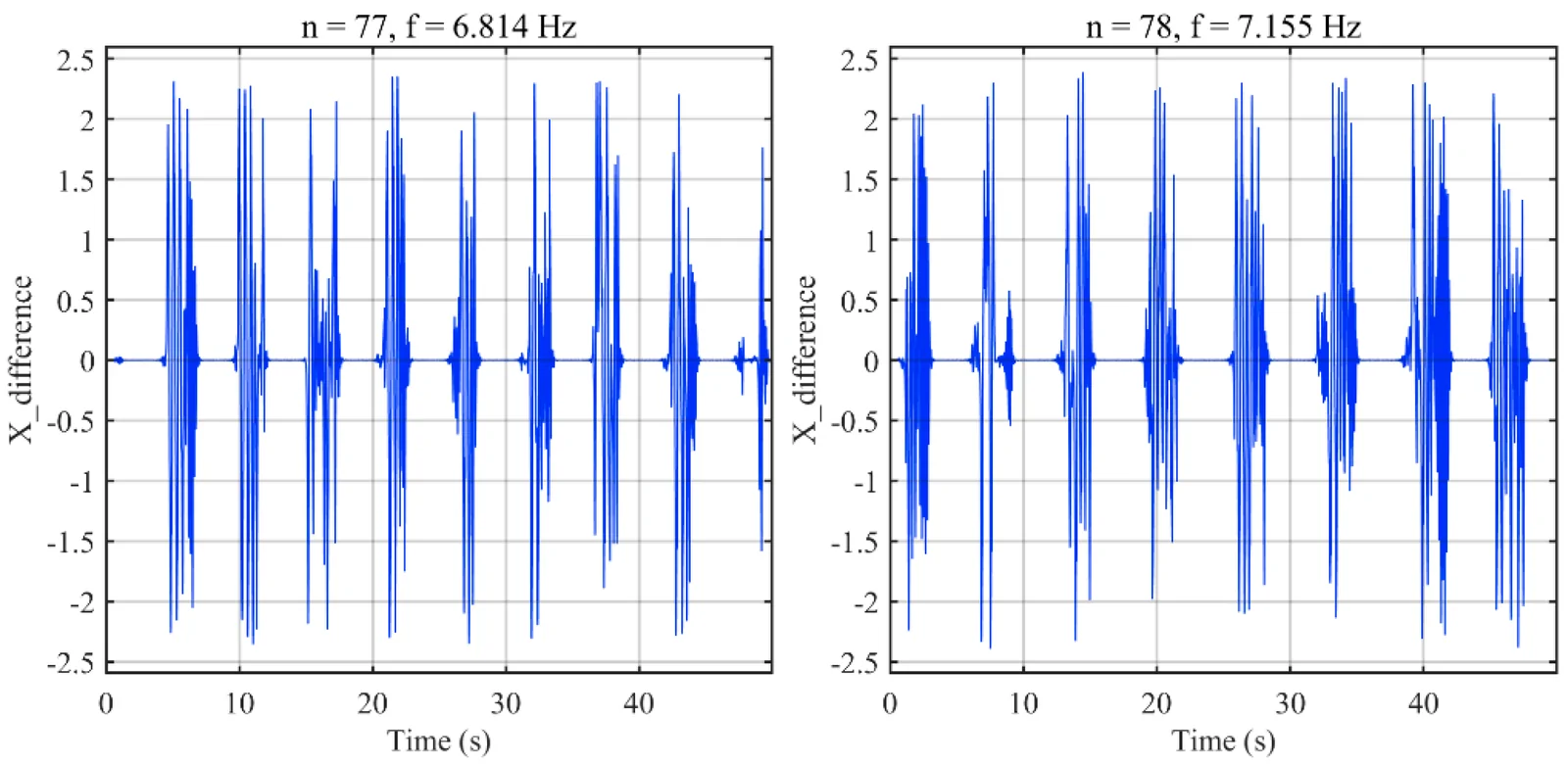
Results of two consecutive occurrences of intermittent chaotic states.

The frequency sweep step parameter,$\;\omega_{n},\;was\;set\;to\;1.05^{n}\omega_{0}$, where$\;\omega_{0}\;=\;1\;\mathrm{rad}/s$. The signal was input into the differential high-order dual-coupled Duffing oscillator system for frequency sweep detection. The results show that the *RCMDE* values of the system output decreased to 0.526 and 0.528, respectively, only during the 77th and 78th frequency sweeps. Compared with the$\;{\mathrm{RCMDE}}_{\mathrm{base}}$ zero baseline value of 0.85, the entropy changes were 0.324 and 0.322, both exceeding the threshold of 0.061, indicating continuous and stable intermittent chaotic phenomena. The corresponding internal driving frequencies were 6.814 and 7.155 Hz, as shown in Figure 13. Based on the internal driving frequencies, the estimated fundamental frequency of the target shaft-rate magnetic field signal was calculated to be 6.985 Hz, with a relative error of 0.21% compared with the actual value of 7 Hz, demonstrating high-frequency estimation accuracy.

This study compares the differential high-order dual-coupled Duffing oscillator detection method with the traditional Duffing oscillator detection method using evaluation metrics, including detection accuracy and false alarm rate. In Monte Carlo simulation experiments, the detection accuracy is the proportion of experiments in which the target is correctly identified in the presence of a shaft-rate magnetic field signal, whereas the false alarm rate is the proportion of experiments where a false target is reported in the absence of such a signal.

For the detection accuracy indicator test, the SNR of the input data were 0, −10, −20, and −30 dB. For each SNR condition, 300 Monte Carlo simulations were conducted. The detection accuracy was determined by calculating the proportion of experiments in which the shaft-rate magnetic field signal was correctly detected. The test results are listed in Table 3. For the false alarm rate indicator test, 300 Monte Carlo simulations were performed. The false alarm rate was obtained by calculating the proportion of experiments where the shaft-rate magnetic field signal was falsely judged to be present. The test results are summarized in Table 4. The simulation results indicate that the proposed differential high-order dual-coupled Duffing oscillator detection method achieved higher detection accuracy and lower false alarm rates than the traditional Duffing oscillator detection method. At a low SNR of −30 dB, the detection accuracy of the differential high-order dual-coupled Duffing oscillator method was 88.33%, while the traditional Duffing oscillator method only achieved a 7.67% detection accuracy.

### 4.2. Marine Experiment Validation

**Figure 14:**
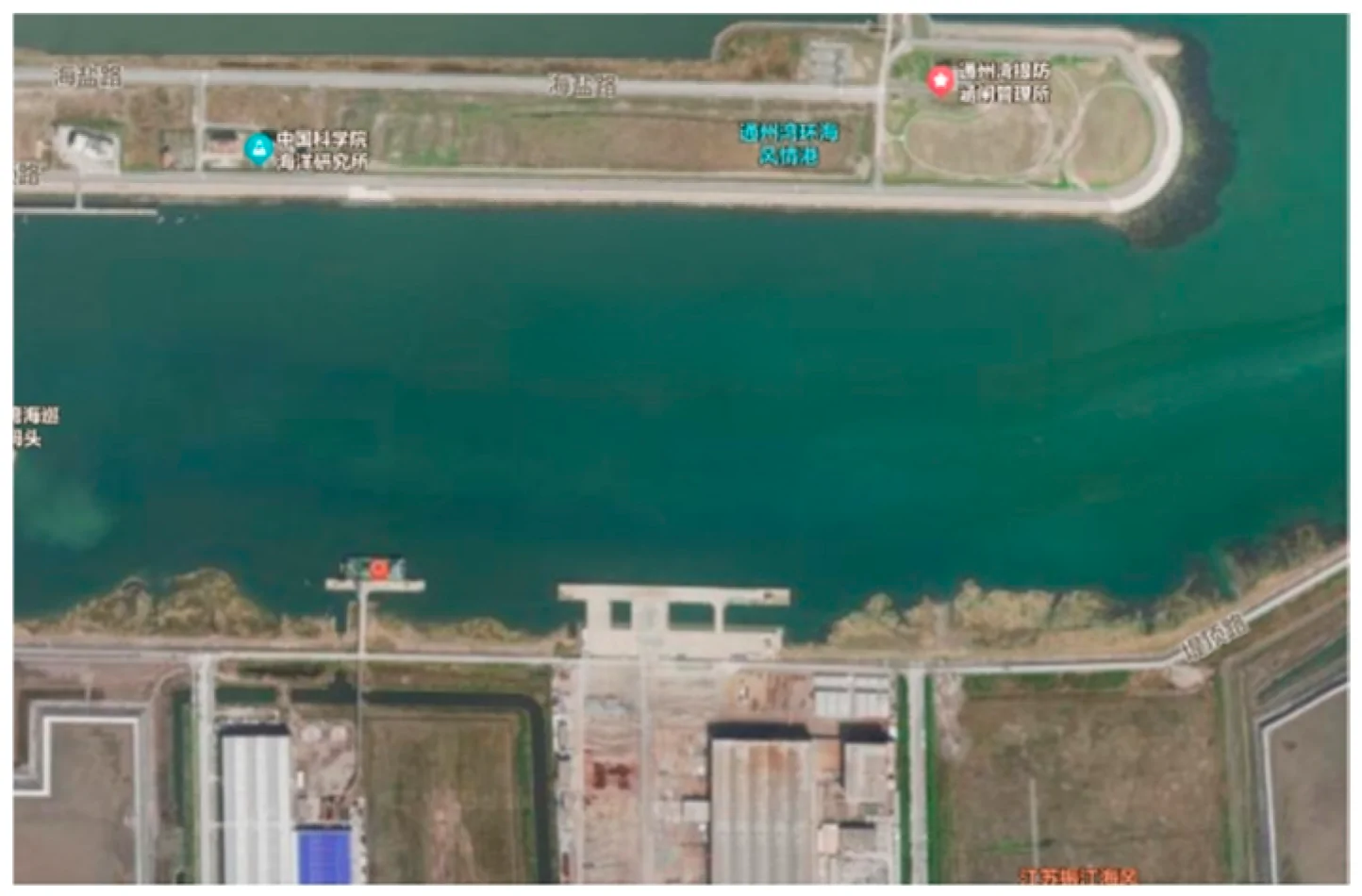
Location of marine experiment.

To further verify the detection capability of the proposed method for shaft-rate magnetic field signals in practical applications, a marine experiment was conducted at the Tongzhou Bay Wharf in Haimen District, Nantong City, Jiangsu Province. The experimental setup is shown in Figure 14.

**Figure 15:**
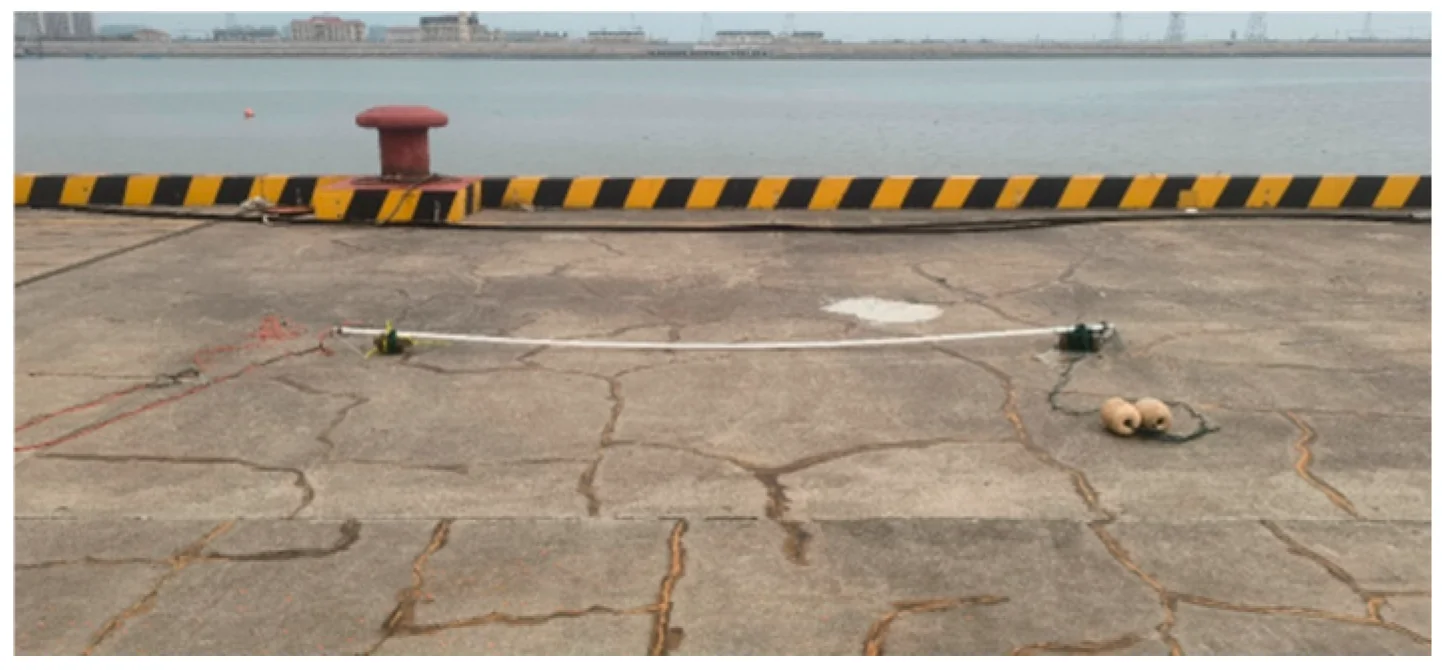
Underwater simulation source of the electric dipole.

**Figure 16:**
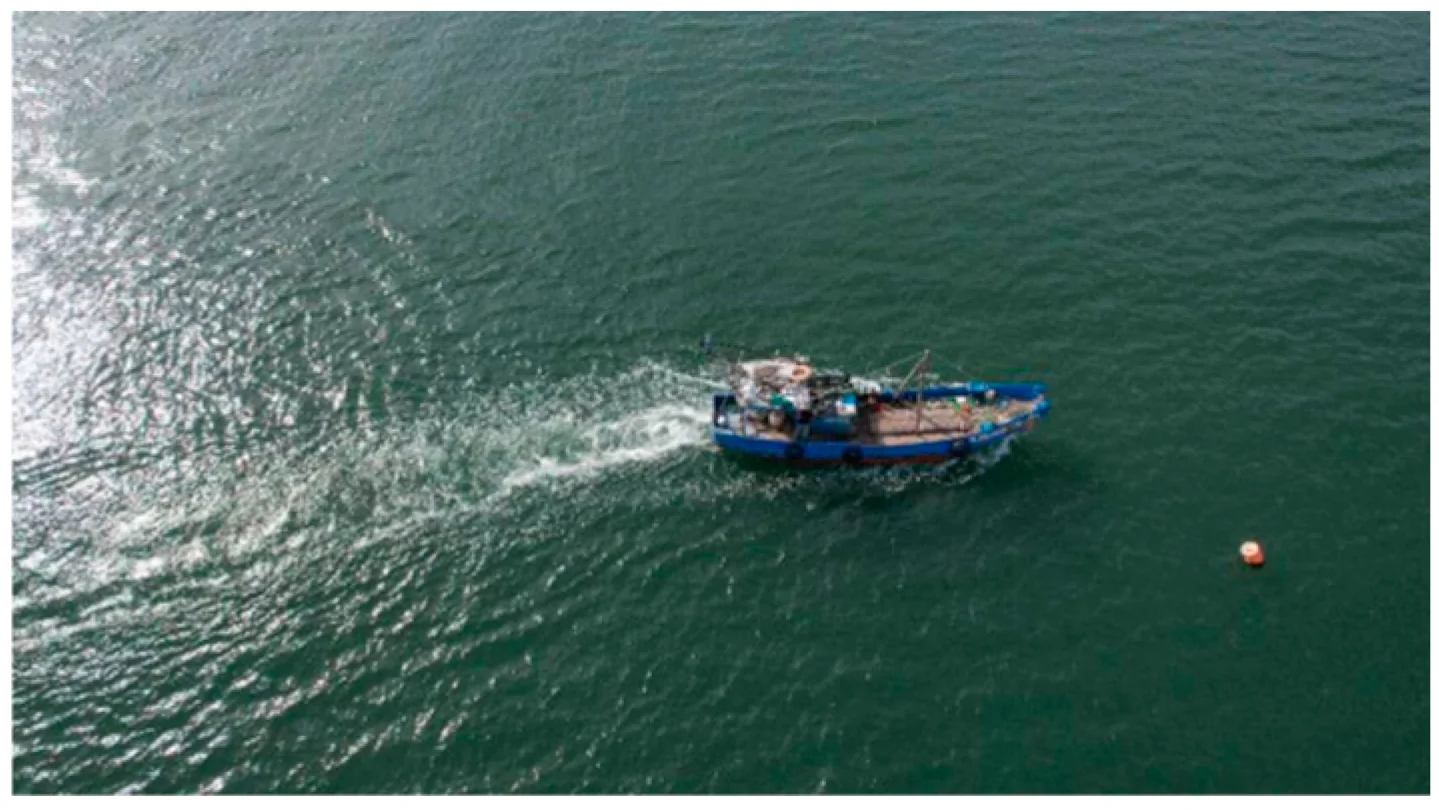
Fishing boat towing the underwater simulation source.

A towed horizontal time-harmonic electric dipole simulation source was used to replace the underwater target. The simulation source employed two platinum alloy electrodes fixed by a 4 m long PVC tube, as illustrated in Figure 15. The simulation source had a frequency of 1.7 Hz, a resistance of 0.1 Ω, a peak voltage of 2 V, and an electric dipole moment calculated as 80 A·m. During the experiment, a fishing boat was used to tow the simulation source underwater. The hull of the fishing boat was treated with anti-rust paint to reduce interference. The electric dipole simulation source and towing vessel are depicted in Figure 16.

**Figure 17:**
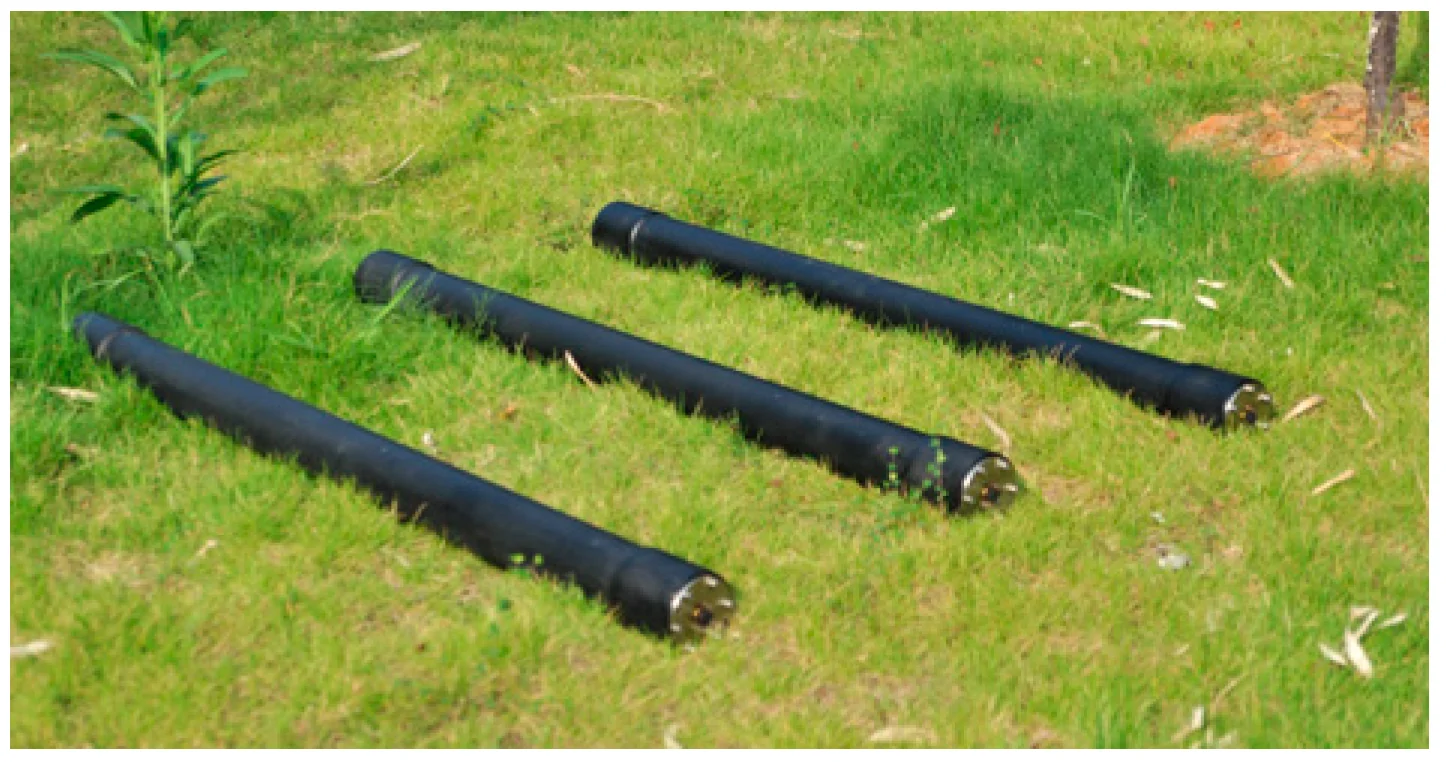
Watertight inductive magnetic field sensor.

During the experiment, the fishing boat towed the simulation source at a constant speed, with the electric dipole simulation source maintained underwater at a depth of 5 m. A watertight seabed uniaxial inductive magnetic field sensor (CMS-01s inductive magnetic sensor from CSSC No. 722 Research Institute, noise $<100\mathrm{fT}/\sqrt{\mathrm{Hz}}@1\mathrm{Hz}$) was used to measure the shaft-rate magnetic field signal. This inductive magnetic field sensor is shown in Figure 17.

**Figure 18:**
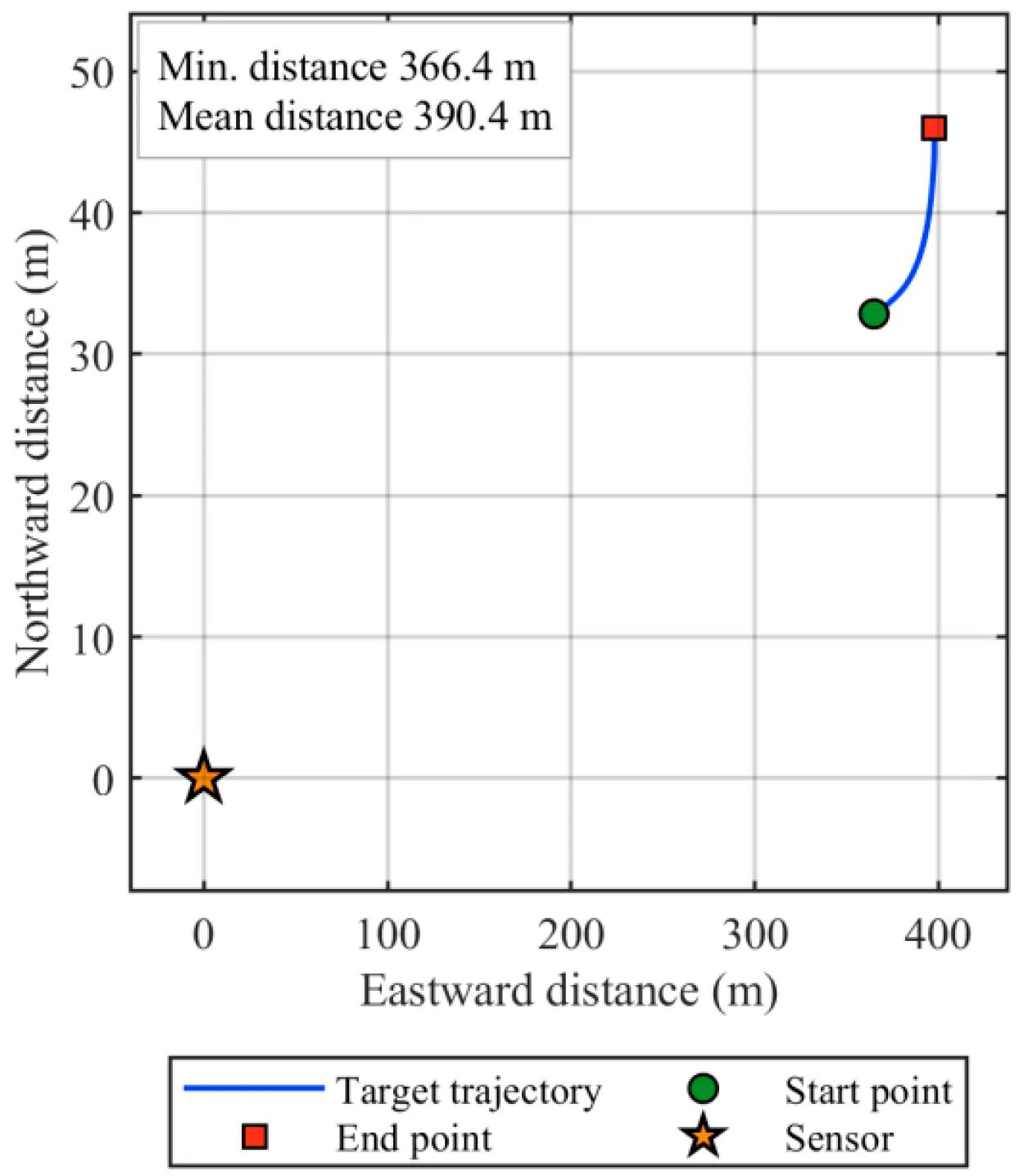
Simulated source navigation trajectory and sensor coordinate diagram.

To verify the effectiveness of the proposed shaft-rate magnetic field detection method, the measured data from the inductive magnetic field sensor corresponding to a segment of the simulated source’s navigation trajectory were selected for analysis. Figure 18 shows the simulated source’s navigation trajectory and the sensor coordinate diagram. It can be observed that the closest distance between the simulated source and the sensor is 366.4 m, and the farthest distance is 390.4 m.

**Figure 19:**
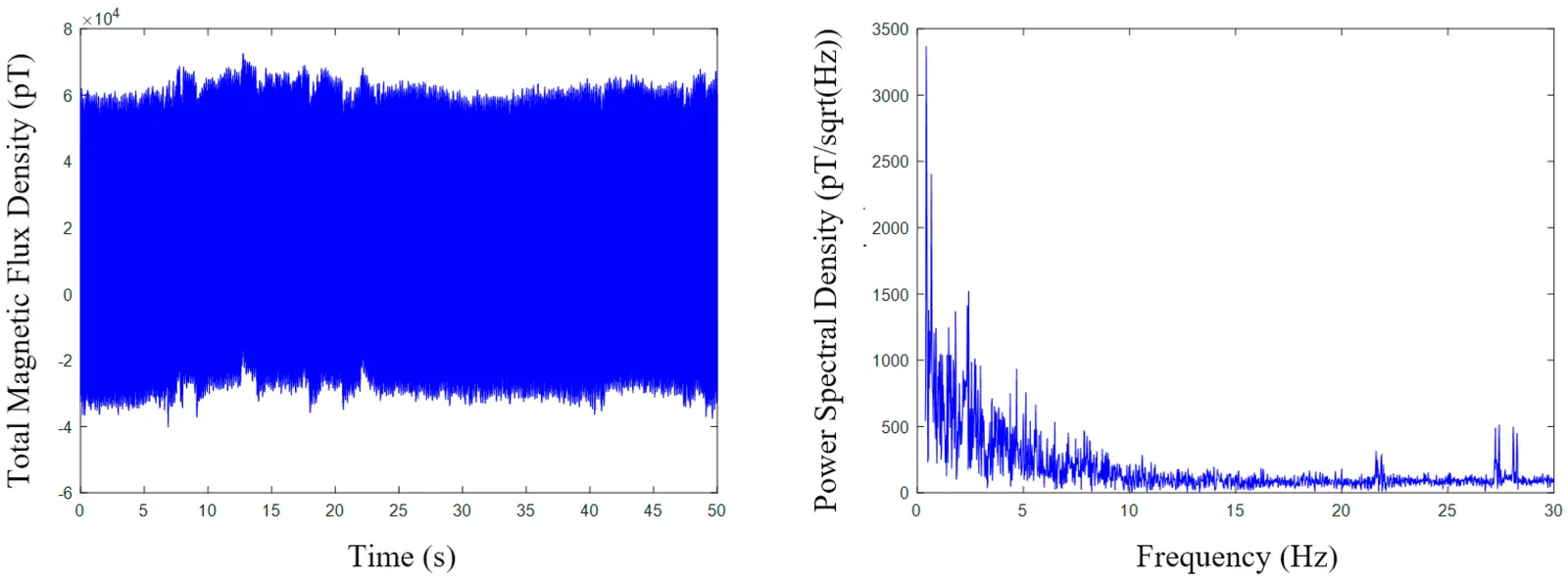
Time-domain waveform and frequency-domain distribution of environmental magnetic field noise data.

**Figure 20:**
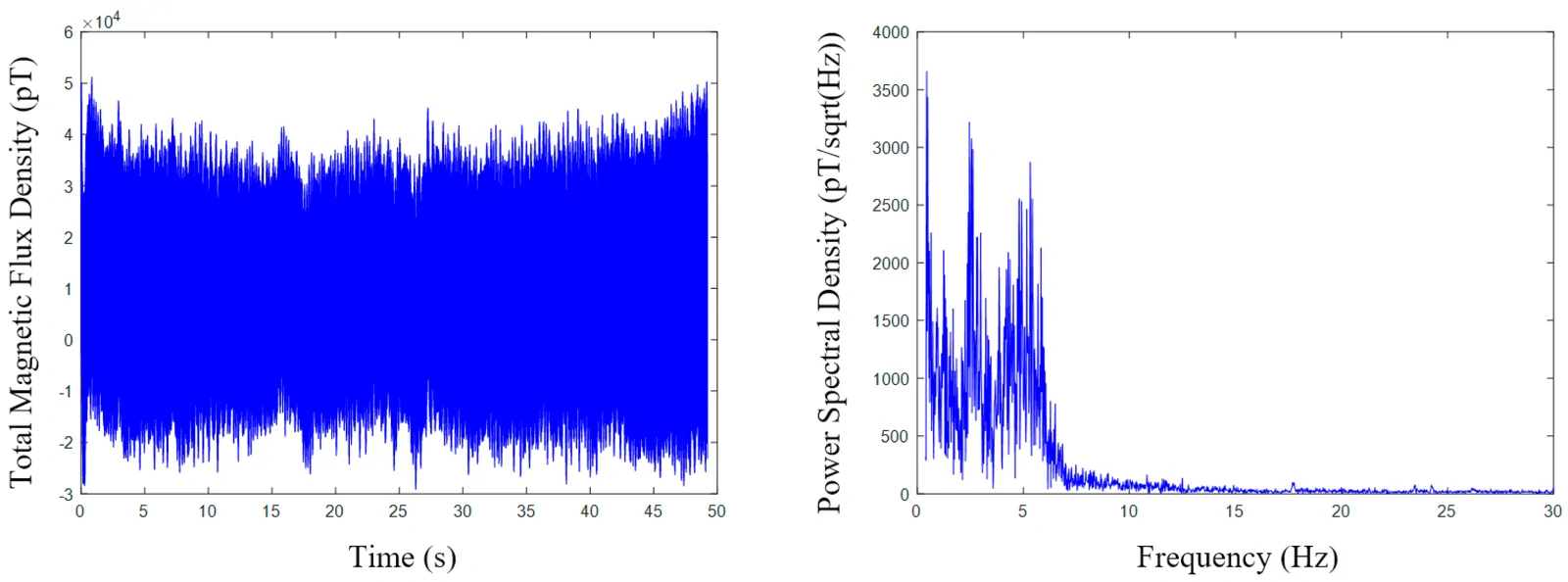
Time-domain waveform and frequency-domain distribution of noisy shaft-rate magnetic field data.

Figure 19 displays the environmental magnetic field noise data collected by the seabed inductive magnetic sensor, while Figure 20 shows the shaft-rate magnetic field data corresponding to Figure 18 collected by the magnetic sensor. It is evident that the environmental magnetic field noise in this marine area is relatively complex with significant background interference, particularly in the low-frequency range. The SNR was only −25.14 dB, posing a considerable challenge for the extraction and identification of shaft-rate magnetic field signals.

**Figure 21:**
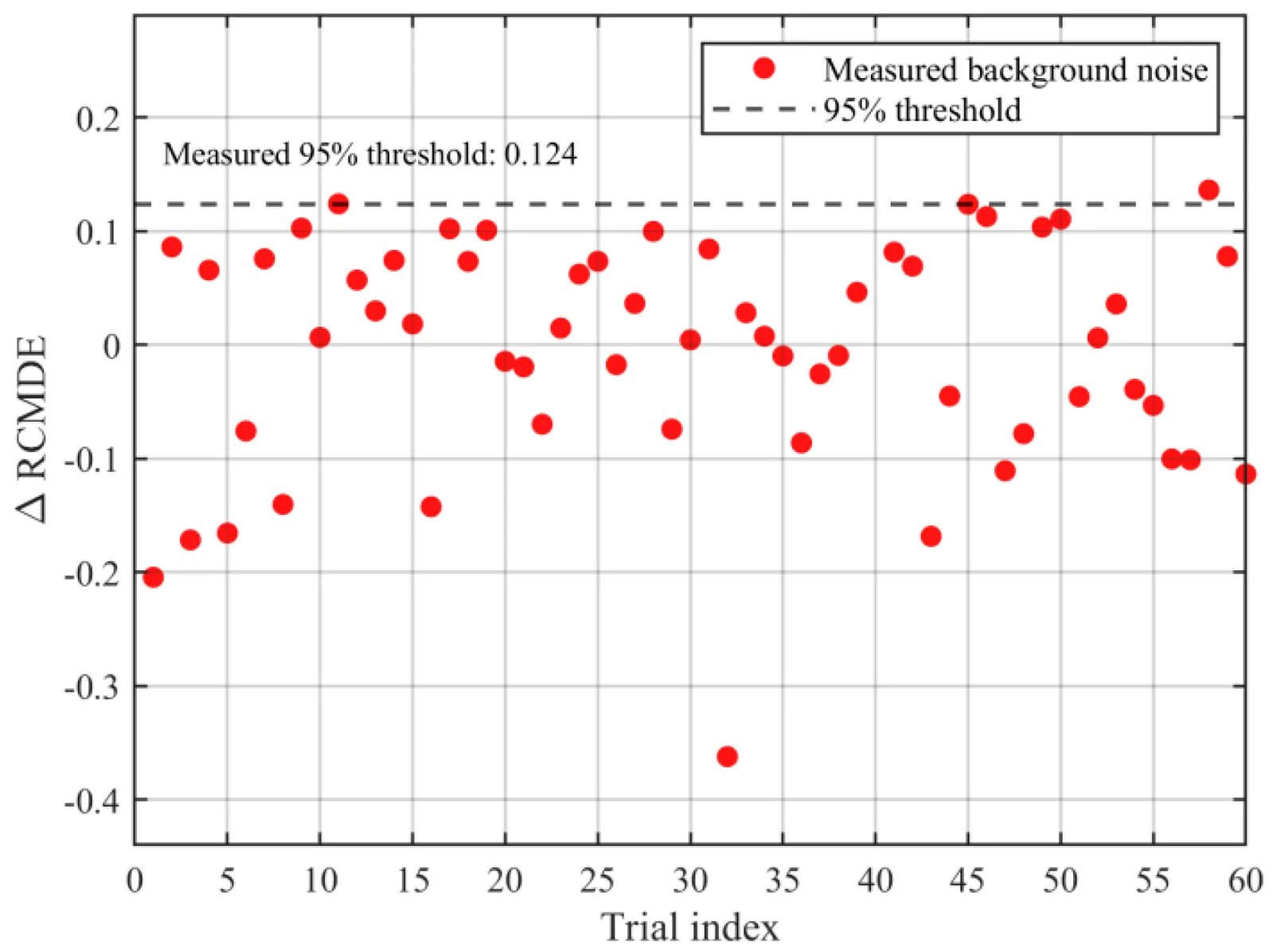
Relative entropy variation characteristics of seabed environmental magnetic field noise data under different conditions.

Next, the differential high-order dual-coupled Duffing oscillator was utilized to detect the shaft-rate magnetic field signals in the marine area. First, the judgment threshold for the intermittent chaotic state was determined. The *RCMDE* values of 50 sets of seabed environmental magnetic field noises were calculated. Then, using their *RCMDE* mean as the zero baseline (calculated as ${\mathrm{RCMDE}}_{\mathrm{base}}\;=\;0.88$), the relative entropy variation characteristics, ΔRCMDE, of the aforementioned 50 sets of data were computed. Finally, the optimal judgment threshold of 0.124 for distinguishing the intermittent chaotic state was derived from Figure 21.

**Figure 22:**
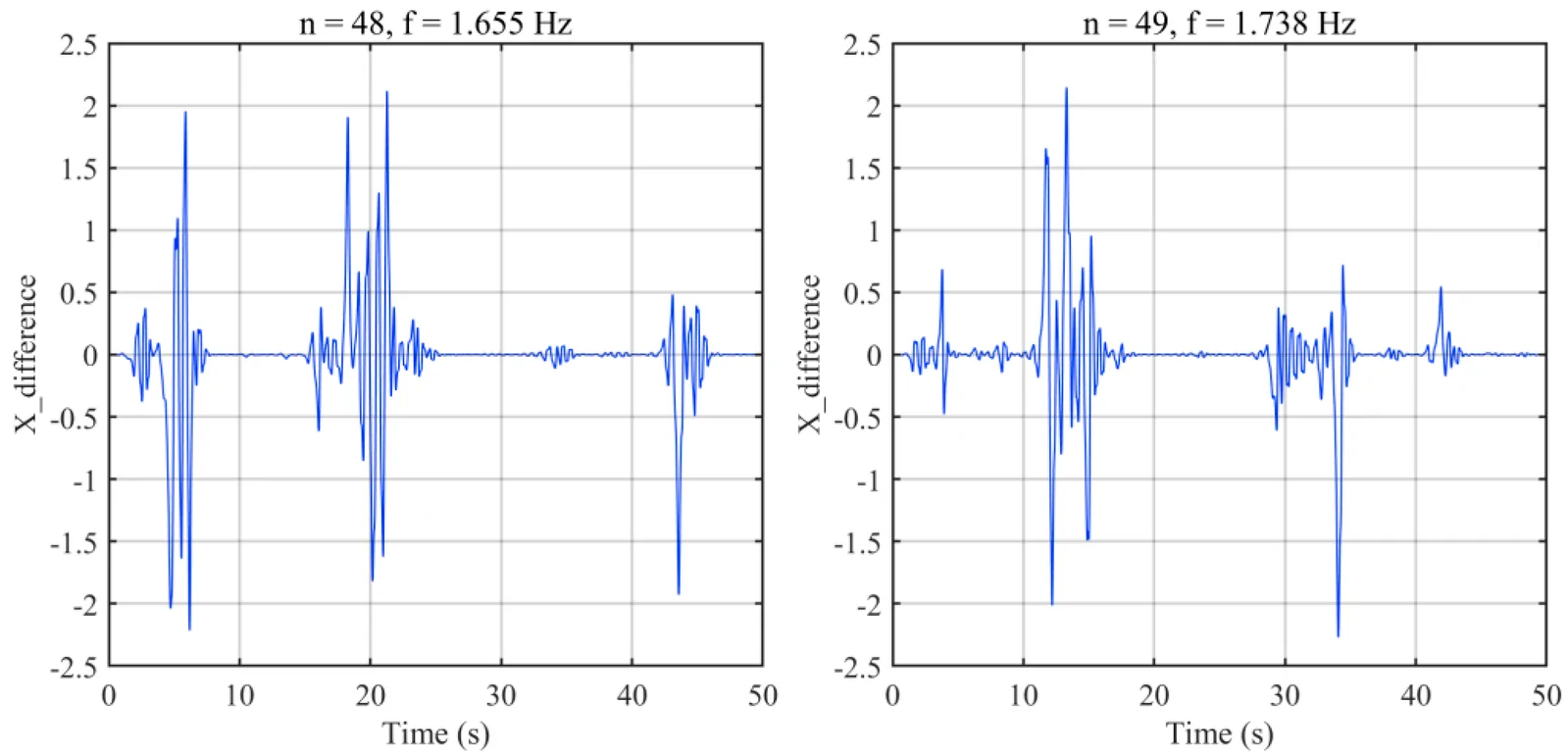
Results showing two successive occurrences of intermittent chaotic states during shaft-rate magnetic field detection.

The sweep step parameter was set as $\omega_{n}\;=\;1.05^{n}\omega_{0}$, where $\omega_{0}\;=\;1\;\mathrm{rad}/s$. The data collected by the seabed inductive magnetic sensor were input into the differential high-order dual-coupled Duffing oscillator system for sweep frequency detection. The results show that the *RCMDE* values of the system output decreased to 0.462 and 0.493, respectively, only during the 48th and 49th sweeps. Compared with the ${\mathrm{RCMDE}}_{\mathrm{base}}$ zero baseline value of 0.88, the entropy changes were 0.418 and 0.387, both exceeding the threshold of 0.124, indicating the occurrence of continuous and stable intermittent chaotic phenomena. The corresponding internal driving frequencies were 1.655 and 1.738 Hz, as shown in Figure 22. Based on the internal driving frequencies, the estimated fundamental frequency of the target shaft-rate magnetic field signal was calculated to be 1.697 Hz, with a relative error of 0.18% compared with the actual value of 1.7 Hz, demonstrating high frequency-estimation accuracy.

Finally, based on the actual datasets from the seabed inductive magnetic sensor, 30 samples containing shaft-rate magnetic field signals were randomly selected, with each group’s data collected over 60 s. A comparison was made between the differential high-order dual-coupled Duffing oscillator detection method and the traditional Duffing oscillator method. The test results are presented in Table 5. The analysis results indicate that the proposed method achieved a detection accuracy rate of up to 93.33%, whereas the traditional Duffing oscillator reached only 36.67%. This demonstrates that the differential high-order dual-coupled Duffing oscillator detection method exhibits excellent performance in detecting weak shaft-rate magnetic field signals in practical engineering scenarios.

## 5. Conclusions

This study addresses the challenge of detecting weak shaft-rate magnetic fields of underwater targets in complex marine environments by proposing a differential high-order dual-coupled Duffing oscillator detection method. By introducing a high-order (third-order) coupling term and a dual-oscillator differential structure, the system’s noise suppression capability and state recognition intuitiveness are enhanced. The designed scale-dispersion joint statistical feature quantity facilitates the determination of the phase-transition threshold of the chaotic system. Combined with an intermittent chaos determination method based on refined composite multiscale dispersion entropy, this approach effectively detects weak shaft-rate magnetic field signals with unknown frequencies and accurately calculates the shaft-rate magnetic field values. Simulation results show that under a low SNR of −30 dB, the detection accuracy of this method for shaft-rate magnetic fields reached 88.33%. Additionally, marine field test results demonstrate that with a measured SNR of −25.14 dB, the detection accuracy was as high as 93.33%, exhibiting significant performance advantages over traditional Duffing oscillator detection methods. This technology can be applied to underwater target detection, large-scale target tracking, and protection of critical ports and underwater infrastructure.

## Figures and Tables

**Table 1:** Electromagnetic parameters of air, seawater, and seabed.

Medium	Permeability (H/m)	Permittivity (F/m)	Conductivity (S/m)
air	$\mu_{0}$	$\varepsilon_{0}$	$\sigma_{0}$
seawater	$\mu_{0}$	$\varepsilon_{1}$	$\sigma_{1}$
seabed	$\mu_{0}$	$\varepsilon_{2}$	$\sigma_{2}$

**Table 2:** Simulation parameters of shaft-rate magnetic field signal.

Parameter	Value
sampling rate	1000 Hz
target speed	5 m/s
target depth	50 m
shaft-frequency base frequency	7 Hz
harmonic order	5
closest point of approach (CPA)	200 m
electric dipole moment	200 A·m
sampling rate	1000 Hz

**Table 3:** Detection accuracy test results of Monte Carlo simulations.

SNR (dB)	Detection Method	Detection Accuracy
0	traditional Duffing oscillator	96.33%
	differential high-order dual-coupled Duffing oscillator	98.33%
−10	traditional Duffing oscillator	80%
	differential high-order dual-coupled Duffing oscillator	98%
−20	traditional Duffing oscillator	38%
	differential high-order dual-coupled Duffing oscillator	98%
−30	traditional Duffing oscillator	7.67%
	differential high-order dual-coupled Duffing oscillator	88.33%

**Table 4:** False alarm rate test results of Monte Carlo simulations.

Detection Method	False Alarm Rate
traditional Duffing oscillator	3.33%
differential high-order dual-coupled Duffing oscillator	2%

**Table 5:** Comparison of detection accuracy.

Detection Method	Detection Accuracy
traditional Duffing system	36.67%
differential high-order dual-coupled Duffing system	93.33%

## Data Availability

The raw data supporting the conclusions of this article will be made available by the authors on request.
